# Diversification processes in Gerp's mouse lemur demonstrate the importance of rivers and altitude as biogeographic barriers in Madagascar's humid rainforests

**DOI:** 10.1002/ece3.10254

**Published:** 2023-07-04

**Authors:** Tobias van Elst, Dominik Schüßler, Romule Rakotondravony, Valisoa S. T. Rovanirina, Anne Veillet, Paul A. Hohenlohe, Jonah H. Ratsimbazafy, Rodin M. Rasoloarison, Solofonirina Rasoloharijaona, Blanchard Randrianambinina, Miarisoa L. Ramilison, Anne D. Yoder, Edward E. Louis, Ute Radespiel

**Affiliations:** ^1^ Institute of Zoology University of Veterinary Medicine Hannover, Foundation Hannover Germany; ^2^ Research Group Vegetation Ecology and Nature Conservation, Institute of Biology and Chemistry University of Hildesheim Hildesheim Germany; ^3^ Ecole Doctorale Ecosystèmes Naturels (EDEN) University of Mahajanga Mahajanga Madagascar; ^4^ Faculté des Sciences, de Technologies et de l'Environnement University of Mahajanga Mahajanga Madagascar; ^5^ Department of Biological Sciences, Institute for Bioinformatics and Evolutionary Studies University of Idaho Moscow Idaho USA; ^6^ Groupe d'étude et de recherche sur les primates (GERP) Antananarivo Madagascar; ^7^ Behavioral Ecology and Sociobiology Unit German Primate Center Göttingen Germany; ^8^ Department of Primate Behavior and Ecology Central Washington University Ellensburg Washington USA; ^9^ Department of Biology Duke University Durham North Carolina USA; ^10^ Grewcock Center for Conservation and Research Omaha's Henry Doorly Zoo and Aquarium Omaha Nebraska USA

**Keywords:** coalescent, diversification, Madagascar, *Microcebus*, phylogeography, RAD sequencing

## Abstract

Madagascar exhibits exceptionally high levels of biodiversity and endemism. Models to explain the diversification and distribution of species in Madagascar stress the importance of historical variability in climate conditions which may have led to the formation of geographic barriers by changing water and habitat availability. The relative importance of these models for the diversification of the various forest‐adapted taxa of Madagascar has yet to be understood. Here, we reconstructed the phylogeographic history of Gerp's mouse lemur (*Microcebus gerpi*) to identify relevant mechanisms and drivers of diversification in Madagascar's humid rainforests. We used restriction site associated DNA (RAD) markers and applied population genomic and coalescent‐based techniques to estimate genetic diversity, population structure, gene flow and divergence times among *M. gerpi* populations and its two sister species *M. jollyae* and *M. marohita*. Genomic results were complemented with ecological niche models to better understand the relative barrier function of rivers and altitude. We show that *M. gerpi* diversified during the late Pleistocene. The inferred ecological niche, patterns of gene flow and genetic differentiation in *M. gerpi* suggest that the potential for rivers to act as biogeographic barriers depended on both size and elevation of headwaters. Populations on opposite sides of the largest river in the area with headwaters that extend far into the highlands show particularly high genetic differentiation, whereas rivers with lower elevation headwaters have weaker barrier functions, indicated by higher migration rates and admixture. We conclude that *M. gerpi* likely diversified through repeated cycles of dispersal punctuated by isolation to refugia as a result of paleoclimatic fluctuations during the Pleistocene. We argue that this diversification scenario serves as a model of diversification for other rainforest taxa that are similarly limited by geographic factors. In addition, we highlight conservation implications for this critically endangered species, which faces extreme habitat loss and fragmentation.

## Graphical abstract

We reconstructed the phylogeographic history of Gerp's mouse lemur (*Microcebus gerpi*) in Madagascar's humid rainforests. Ecological niche models and genomic results jointly suggest that rivers acted as biogeographic barriers depending on both their size and the elevation of headwaters. This species likely diversified through repeated cycles of dispersal punctuated by isolation to refugia during Pleistocene paleoclimatic fluctuations.

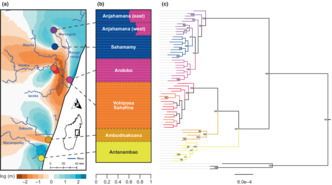

## 1 INTRODUCTION

The island of Madagascar has been separated from other land masses for more than 80 million years (Pande et al., 2001; Plummer & Belle, 1995; but see Masters et al., 2020), allowing a unique flora and fauna to evolve. It is one of the most biologically diverse places on earth and exhibits exceptionally high levels of endemism across taxonomic levels in both plants and animals (Estrada et al., 2017; Goodman & Benstead, 2005). Due to a large number of endemic radiations, congruent biogeographic patterns across taxa and pronounced environmental gradients, Madagascar is a promising system to study the drivers of the evolution of biodiversity in tropical ecosystems (Vences et al., 2009). Madagascar's ecosystems are also severely threatened by the loss and fragmentation of natural vegetation structures (Estrada et al., 2017; Morelli et al., 2020; Schwitzer et al., 2013), making it a major biodiversity hotspot and conservation priority (Ganzhorn et al., 2001; Myers et al., 2000).

Models to explain the diversification and distribution of species in Madagascar and the tropics more generally have stressed the importance of historical variability in climate conditions, particularly during the Pleistocene. Specifically, models of lineage diversification have identified changing water and habitat availability depending on altitude as a potential driver of speciation (reviewed in Brown et al., 2014; Hewitt, 2000; Vences et al., 2009). It is generally assumed that the island's climate underwent cycles of cooler dry and warmer humid conditions that were linked to the global temperature fluctuations associated with alternating glacial and interglacial periods in the Quaternary (Burney et al., 2004; deMenocal, 2004; Ehlers & Gibbard, 2011; Gasse & Van Campo, 2001; Snyder, 2016; Teixeira, Montade, et al., 2021). Hypotheses such as the riverine barrier (Craul et al., 2007; Goodman & Ganzhorn, 2004; Martin, 1972) and retreat‐dispersal watershed models (see also Eco‐Geo‐Clim model; Mercier & Wilmé, 2013; Wilmé et al., 2006) argue that forest habitats were likely widespread and continuous during warm and humid conditions coinciding with interglacials, facilitating high connectivity for forest‐adapted species over large distances and providing corridors to cross riverine barriers at higher elevation headwater regions. In contrast, forests likely contracted to isolated refugia during cooler and more arid conditions (Burney et al., 1997; Gamisch et al., 2016; Gasse & Van Campo, 2001; Kiage & Liu, 2016), with the consequence that an expanding open arid landscape or rivers could no longer be crossed. Refugial populations are hypothesized to have evolved in allopatry with increasing genetic differentiation, thus leading to reproductive isolation over time.

The relative importance of the proposed biogeographic models for the diversification of the various forest‐adapted taxa of Madagascar with differing life history strategies and dispersal abilities has yet to be understood. For instance, rivers delimit species distributions and determine population structure in some lemur taxa (e.g., Craul et al., 2007; Pastorini et al., 2003) but not in others (e.g., Craul et al., 2008; Sgarlata et al., 2018). Accordingly, more empirical work is needed to identify how and why the evolutionary trajectory of different lineages has been shaped by different types of barriers. Genome‐scale phylogeographic studies are a valuable tool to quantify genetic variation in a spatial and temporal context with unprecedented confidence and resolution. Such studies allow the identification of genetic patterns and the development of informed hypotheses of the evolutionary processes that may be responsible for the observed patterns (Berv et al., 2021; Corbett et al., 2020; Poelstra et al., 2018; Tiley et al., 2022).

Mouse lemurs of the genus *Microcebus* (Cheirogaleidae) are particularly well‐suited for modeling diversification processes of forest‐adapted mammals in Madagascar. The mouse lemur radiation comprises at least 24 cryptic, nocturnal species that can be found in all forest habitats and bioclimatic zones of Madagascar and many of which are restricted to narrow geographic ranges (microendemism) (Hotaling et al., 2016; Mittermeier et al., 2010; Poelstra et al., 2021; Schüßler, Blanco, et al., 2020). Due to their small size, short generation time and high habitat specificity (Mittermeier et al., 2010), it can be hypothesized that they are particularly sensitive to the aforementioned geographic barriers and that genomic signatures of cyclic geographic isolation will manifest rapidly. Analyses by Poelstra et al. (2021) suggest that the diversification of mouse lemurs occurred relatively recently during the Pleistocene (but see Everson et al., 2023; Herrera & Dávalos, 2016; Louis & Lei, 2016), and previous studies have already indicated that rivers (Martin, 1972; Olivieri et al., 2006; Pastorini et al., 2003; Tiley et al., 2022), watersheds (Mercier & Wilmé, 2013; Wilmé et al., 2006) and paleoclimatic fluctuations (Blair et al., 2014; Poelstra et al., 2021; Teixeira, Montade, et al., 2021; Teixeira, Salmona, et al., 2021) were significant determinants of mouse lemur population structure and demography, but these were only rarely modeled in an integrative way.

**FIGURE 1:**
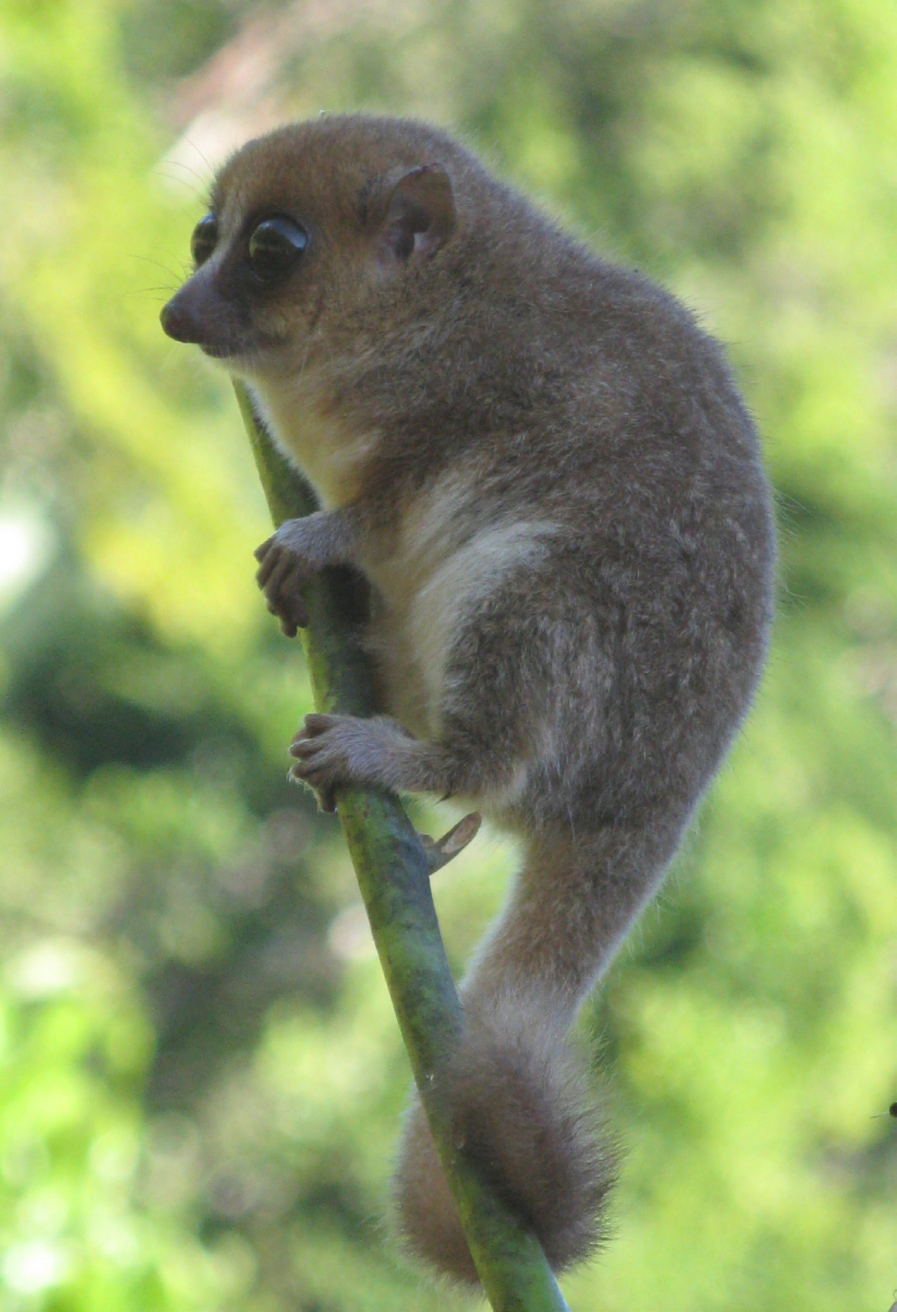
*Microcebus gerpi* at Sahafina in 2009.

The present study aims to explore how rivers, elevation and paleoclimate interacted to generate population structure and genetic differentiation in the critically endangered microendemic Gerp's mouse lemur (*Microcebus gerpi*; Figure 1). This poorly studied species is a promising candidate to identify the role of these drivers of diversification given that its distribution in the lowland rainforests of Madagascar's east coast is separated into multiple inter‐river systems (IRSs) with a complex altitudinal profile (Andriaholinirina et al., 2014; Radespiel et al., 2012). We significantly expand the sampling of *M. gerpi* and use restriction site associated DNA (RAD) markers to reconstruct its phylogeographic history. Applying population genomic and coalescent‐based techniques, we estimate genetic diversity, population structure, gene flow and divergence times among *M. gerpi* populations and its two sister species *M. jollyae* and *M. marohita*. We combine our genomic results with ecological niche models to better understand the relative barrier function of rivers and altitude. Finally, we discuss the conservation implications of our study for *M. gerpi*.

## 2 MATERIALS AND METHODS

### 2.1 Study sites and sampling

**FIGURE 2:**
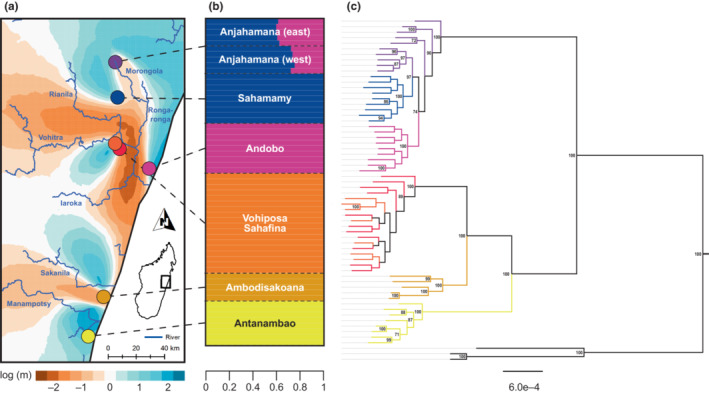
Phylogeography of *Microcebus gerpi* in eastern Madagascar. (a) Sampling locations of *M. gerpi* populations (colored dots) and estimated effective migration surface using 1000 demes. Effective migration rate is given on log_10_ scale. (b) Admixture proportions (horizontal bars) for individuals of *M. gerpi* populations. Shown are results of the best‐scoring likelihood model of 10 independent ngsadmix runs using five a priori clusters, which illustrate the admixed ancestry of individuals at Anjahamana. Log‐likelihoods and Δ*K* are given in Figure S5. (c) Maximum likelihood tree inferred with raxml‐ng. Branch and tip colors correspond to populations in a. Black tips represent *M. jollyae* and *M. marohita* outgroups. Bootstrap values are given if above 70%. Scale is substitutions per site.

The study area is located between the Ivondro and the Mangoro rivers on Madagascar's east coast (Figure 2a), which are the distributional boundaries of the two mouse lemur species adjacent to *Microcebus gerpi*, *M. simmonsi* and *M. marohita*, respectively (Louis et al., 2006, 2008; Rasoloarison et al., 2013). To the west, the region is demarcated by Madagascar's central highlands, with a steep elevational gradient towards the coast. Rivers of different size and headwater height separate the region into multiple inter‐river systems (IRS). Due to high levels of deforestation, continuous forest tracts are restricted to higher elevations and only tiny fragments remain in lowland areas (Vieilledent et al., 2018). Forests at higher altitudes seem to be exclusively populated by *M. lehilahytsara*, with no reported sightings of *M. gerpi* (Radespiel et al., 2012; Roos & Kappeler, 2006; Tiley et al., 2022).

A total of 62 *Microcebus gerpi* individuals were sampled via ear biopsies between August and December 2018 at seven lowland rainforest sites in the area (hereafter referred to as populations), including the type locality at Sahafina (Radespiel et al., 2012; Figure 2a, Appendix S1: Table S1). At one site, Anjahamana, individuals were collected both east and west of the adjacent Morongola river, which is about 5–10 m wide there. We also included five *M. gerpi*, two *M. jollyae* and two *M. marohita* individuals available from prior field work. RAD sequences of three *M. murinus* individuals from Poelstra et al. (2022) were added as outgroups. Collection information is given in Table S1.

### 2.2 RAD sequencing, genotyping, and locus extraction

We generated RADseq libraries following two single‐digest *Sbf*I protocols (see Table S2). Raw RAD reads were demultiplexed with process_radtags of stacks v2.0b (Rochette et al., 2019), trimmed with trimmomatic v0.39 (Bolger et al., 2014), and aligned against the *Microcebus murinus* reference genome (Mmur 3.0; Larsen et al., 2017) with BWA‐MEM (Li & Durbin, 2009). Using samtools v1.11 (Li et al., 2009), reads not mapping to autosomal scaffolds or with a mapping quality below 20 were removed. Paired‐end reads were filtered for proper pairing and deduplicated. We estimated the number of RAD loci sequenced for each individual and locus coverage as the forward read depth at the respective *Sbf*I cutting site.

Genotypes were called from BAM files with GATK v4.1.9.0 (McKenna et al., 2010) according to the gatk best practices workflow (GATK Team, 2021). After removing indels, FS6 filtering recommendations of O'Leary et al. (2018) were applied with modified thresholds (see Appendix S2: Supplementary methods) using vcftools v0.1.17 (Danecek et al., 2011). Because RAD libraries were sequenced with low anticipated coverage, we additionally estimated genotype likelihoods with the samtools model in angsd v0.934 (Korneliussen et al., 2014), which allows incorporating information about uncertainty in genotype calls. We applied the same filtering as in Poelstra et al. (2021) and excluded outgroup individuals and those not passing FS6 filtering (see supplementary methods). In addition, minor allele frequency (MAF) spectra of each population pair were inferred with realsfs of ANGSD from genotype likelihoods. We also produced FASTA files for phased RAD loci, using the pipeline of Poelstra et al. (2021) (see supplementary methods). Extracted RAD loci were aligned with MUSCLE v3.8.31 (Edgar, 2004) and concatenated with AMAS v1.0 (Borowiec, 2016) for subsequent phylogenetic inference. An overview of created SNP sets can be found in Table S3.

### 2.3 Phylogenetic inference

We used three approaches for phylogenetic inference to reveal potential phylogenetic conflict. First, maximum likelihood (ML) inference was performed on the unpartitioned concatenated alignment using the GTR + Γ substitution model and the stamatakis method for ascertainment bias correction in raxml‐ng v1.0.2 (Kozlov et al., 2019). We conducted 20 unconstrained ML searches with 100 bootstrap replicates. Second, svdquartets (Chifman & Kubatko, 2014) implemented in PAUP* v4.0a (build 168) (Swofford, 2003) was used for phylogenetic inference from RAD loci under the multispecies coalescent model. We evaluated all quartets with 100 standard bootstrap replicates and assigned individuals as tips. Third, we estimated pairwise genetic distances from the concatenated alignment and built a split network with the neighbornet method (Bryant & Moulton, 2004) in splitstree v4.17.1 (Huson & Bryant, 2006).

### 2.4 Population structure

Population structure was inferred from genotype likelihoods via model‐based and model‐free approaches. First, we performed principal component analysis with pcangsd (Meisner & Albrechtsen, 2018). Second, we inferred individual ancestries with ngsadmix (Skotte et al., 2013), assuming one to ten a priori clusters (*K*). Third, we estimated weighted fixation indices (*F*_*ST*_; Reynolds et al., 1983) from joint MAF spectra with realsfs and genetic distances between individuals from genotype calls with the R package ‘vcfR’ v1.12 (Knaus & Grünwald, 2017). To test the presence of isolation‐by‐distance, pairwise *F*_*ST*_ and mean genetic distances (between populations) were input to Mantel tests against geographic distances, using 9999 permutations in the R package ‘vegan’ v2.5–7 (Oksanen et al., 2020). Finally, connectivity between populations was visualized with estimated effective migration surfaces (EEMS) (Petkova et al., 2015) based on an average genetic dissimilarity matrix estimated from genotype calls with bed2diffs v1. EEMS was run for 4,000,000 generations with a burn‐in of 1000,000, using three alternative numbers of demes (200, 500, 1000).

### 2.5 Coalescent modeling

The coalescent sampler g‐phocs (Gronau et al., 2011) was used to infer divergence times, effective population sizes (*N*_*e*_), and migration rates between recent lineages from extracted loci. In our models, we included *M. jollyae*, *M. marohita*, and all *M. gerpi* populations except Anjahamana, which was recovered as non‐monophyletic in phylogenetic inference. Similarly, the populations Vohiposa and Sahafina were combined because they were not reciprocally monophyletic. For computational reasons, we used a subset of two individuals per population (see Table S1), which is sufficient to estimate coalescent parameters (Huang et al., 2020). We fixed the topology following results of phylogenetic inference. We first ran exploratory models with single bidirectional migration bands between lineages for which gene flow would be geographically feasible (representing continuous gene flow since the divergence of those populations). Subsequently, the final model was built with all migration bands whose 95% highest posterior density (HPD) interval did not overlap with zero. Four replicates of the final model and of a model with no migration bands were run for 2,000,000 generations with a burn‐in of 10%. Convergence of chains and effective sample size (ESS) were checked with tracer v1.7.2 (Rambaut et al., 2018).

To account for uncertainty surrounding mouse lemur generation times and mutation rates (Campbell et al., 2021; Radespiel et al., 2019; Yoder et al., 2016; Zohdy et al., 2014), posterior distributions of coalescent units (*θ*, *τ*, *m*) were converted to effective population size (*N*_*e*_), divergence time in years and population migration rate by drawing estimates of generation time from a lognormal distribution with mean ln(3.5) and standard deviation ln(1.16) and estimates of mutation rate from a gamma distribution with mean 1.236 × 10^−8^ and variance 0.107 × 10^−8^ (see supplementary methods; Poelstra et al., 2021).

Finally, the genealogical divergence index (*gdi*) (Jackson et al., 2017) was calculated as $\mathrm{gdi}=1-e^{\frac{-2*\tau }{\theta }}$ (Leaché et al., 2019) from posterior estimates of the model without migration to compare levels of divergence within *M. gerpi* to those between *M. marohita* and *M. jollyae*. Because *θ* can refer to either of the two divergent lineages, the *gdi* was estimated twice for each node. As a rule of thumb, values below 0.2 indicate intraspecific differentiation and values above 0.7 suggest species‐level divergence (ambiguous zone: 0.2 < *gdi* < 0.7).

### 2.6 Ecological niche modeling

We developed ecological niche models for *M. gerpi* to better assess which bioclimatic and geographic features impose barriers to connectivity and gene flow. We assembled occurrence records for *M. gerpi* and *M. lehilahytsara* (a likely competitor in higher elevation forests) from our own fieldwork and the literature (Andriamasimanana et al., 2001; Radespiel et al., 2012; Rakotondratsimba et al., 2013; Ratsimbazafy et al., 2013; Yoder et al., 2016) (Table S4). After rarefication to reduce spatial bias (Boria et al., 2014), 13 and 12 presence records remained for *M. gerpi* and *M. lehilahytsara*, respectively. We employed two alternative approaches to model suitable habitats for *M. gerpi*. First, we used the maxent algorithm (Phillips et al., 2006) in the R package ‘ENMTools’ v1.0.6 (Warren et al., 2021) to model presence‐only data against a randomly generated background. Second, we applied a random forest model constructed on presence‐absence data in the R package ‘biomod2’ v3.5.1 (Thuiller et al., 2009), using *M. lehilahytsara* occurrences as absence records for *M. gerpi*. Models were validated with the R package ‘ENMeval’ v2.0.3 (Kass et al., 2021) using the Continuous Boyce Index (CBI) and Area Under the Curve (AUC) with a k‐1 Jackknife procedure. As predictors, we used 19 bioclimatic variables obtained from the CHELSA database v2.1 (Karger et al., 2017). Variables were first clipped to the study region and then transformed using PCA with the R packages ‘raster’ v3.5–21 (Hijmans, 2022) and ‘RStoolbox’ v0.3.0 (Leutner et al., 2022). The first three PCs explained 93.9% of the total variation and were therefore used for modeling to handle multicollinearity of the bioclimatic variables and to not overfit ecological niche models.

## 3 RESULTS

### 3.1 RADseq statistics

We obtained an average of 9,105,386 raw reads per individual. After trimming, filtering and reference alignment, 36.48% of the reads remained, covering 69,081 RAD loci with a mean F1 coverage of 12.27×. Detailed sequencing statistics are given in Table S2. Nine individuals were removed during filtering, leading to a total of 59 *M. gerpi* and six outgroup individuals. Among these, 312,924 variant sites were identified that passed filters (Table S3). Considering only *M. gerpi* individuals, 226,115 and 232,256 variant sites were retained after filtering genotype calls and genotype likelihood estimates, respectively. Percentage of missing data per individual is given in Table S5. Extraction of full locus sequences and subsequent filtering recovered 7332 loci with a mean length of 649 bp, leading to a concatenated alignment of 4,757,823 bp (83,512 parsimony‐informative sites) with 4.06% missing data (Tables S6 and S7).

### 3.2 Phylogenetic inference

All three approaches showed a deep split between *M. gerpi* populations north and south of the Rianila river (Figure 2c, Appendix S2: Figures S1–S3). The northern populations Sahamamy and Andobo formed well‐supported monophyletic clades in all analyses. This was not the case for Anjahamana, which was only monophyletic in quartet‐based but not in ML inference and showed high reticulation in the split network. Anjahamana individuals sampled east of the Morongola river clustered as sister to Sahamamy whereas individuals sampled west were grouped at the base of the northern clade. Phylogenetic relationships among populations south of the Rianila were congruent across all analysis. The populations Antanambao and Ambodisakoana were monophyletic with high support, with some minor reticulation indicated by the split network. Individuals of the geographically close populations Sahafina and Vohiposa formed a mixed clade, which was sister to Ambodisakoana.

### 3.3 Population structure

Analyses of population structure were congruent among each other and supported findings of phylogenetic inference. Principal component analysis clearly separated populations north and south of the Rianila along PC1 (54.5% of variation; Figure S4). Except for Vohiposa and Sahafina, southern populations were well‐separated along PC2 (17.04% of variation). Ambodisakoana took an intermediate position between Antanambao and Vohiposa/Sahafina, mirroring its geographic location. Similar to phylogenetic inference, no separation was evident between northern populations.

The most likely number of clusters for the inference of individual ancestries was *K* = 3 (Figure S5), corresponding to (1) populations north of the Rianila, (2) Sahafina/Vohiposa, and (3) Antanambao (Figure S6). Ambodisakoana received mixed ancestry from the second and third cluster. Support for *K* = 2 was similarly high, separating populations north and south of the Rianila. Increasing *K* led to relatively clear division into defined *M. gerpi* populations, with relatively high support for *K* = 5, where Anjahamana individuals received mixed ancestry from Sahamamy and Andobo (Figure 2b, Figures S6–S8). Anjahamana individuals east of the Morongola had slightly higher Andobo ancestry (~0.4) than those west of the river (~0.3).

**FIGURE 3:**
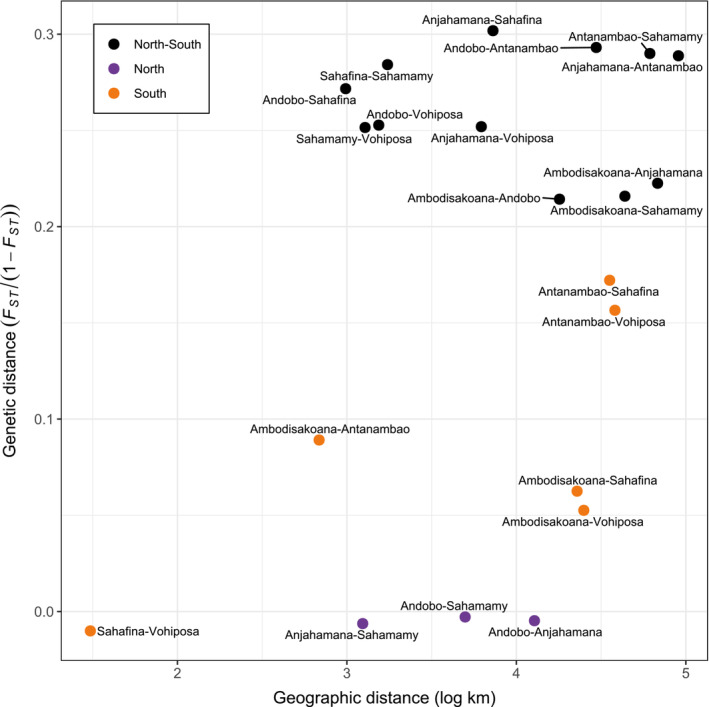
Slatkin's linearized, weighted *F*_ST_ values between *Microcebus gerpi* populations plotted against geographic distances (in log km). Fixation indices were calculated with realsfs. Geographic distances between populations were calculated as means between individual distances. Colors indicate whether the compared populations were north and south of (black), both north of (purple), or both south of the Rianila river (yellow).

Pairwise *F*_*ST*_ values were highest when comparing populations north and south of the Rianila (Figure 3, Table S8; geographic distances are given in Tables S9 and S10). The lowest differentiation was found among northern populations and between Sahafina and Vohiposa. South of the Rianila, Antanambao exhibited relatively high *F*_*ST*_ values to any other population. Differentiation between Ambodisakoana and Sahafina or Vohiposa, respectively, was comparably low considering the large geographic distance. Genetic distances between individuals mirrored these findings (Figure S9, Tables S11 and S12). No significant pattern of isolation‐by‐distance was revealed by Mantel tests (*F*_*ST*_: *r* = .2987, *p* = .1187; genetic distances: *r* = .3961, *p* = .0520), indicating that factors other than distance explain the observed genetic structure.

Estimated effective migration surfaces were largely congruent across number of demes and mirrored findings of other population structure analyses (Figure 2a, Figure S10). They supported a lack of gene flow between populations north and south of the Rianila, as a highly negative log migration rate was inferred along the river. A migration barrier was also found along the Morongola and Rongaronga rivers, which suggests that migration among northern populations occurred predominantly over the headwaters of the Morongola. South of the Rianila, negative log migration rates were mainly estimated between the Sakanila and Manampotsy rivers but sampling there was likely too limited for confident inference.

### 3.4 Coalescent modeling

**FIGURE 4:**
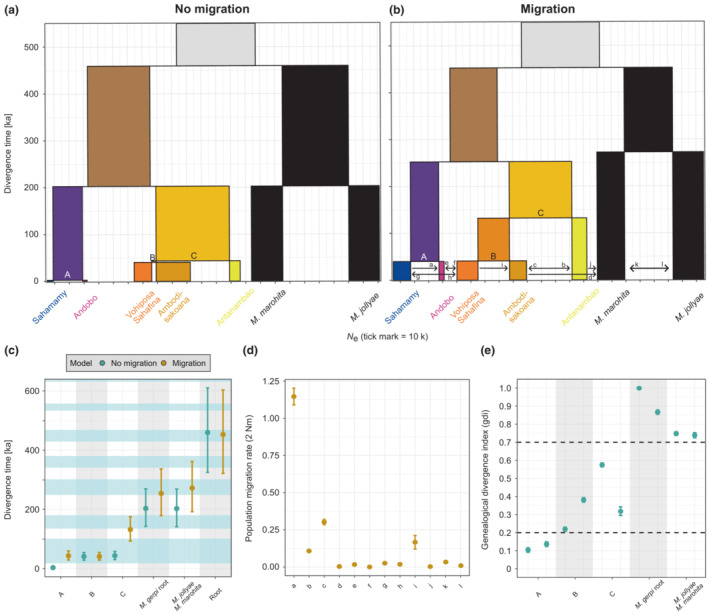
Demographic histories of *Microcebus gerpi* populations, *M. jollyae*, and *M. marohita* inferred by g‐phocs under models with and without migration. All estimates are based on mean posterior distributions across four replicate runs. (a) Divergence times (*y*‐axis) and effective population sizes (*x*‐axis) under a model without migration. (b) Divergence times, effective population sizes and significant migration bands under a model with migration. (c) Divergence times with 95% highest posterior density (HPD) interval at each node for both models. Majuscules refer to a and b. Glaciation periods according to EPICA Community Members (2004) are indicated by blue shading. (d) Population migration rate (*2Nm*) with 95% HPD interval for significant migration bands. Minuscules refer to b. (e) Genealogical divergence index (*gdi*) with 95% HPD interval based on the model without migration. Two estimates are given per node because the *gdi* depends on *θ* which can refer to either of the two divergent lineages. Majuscules refer to a and b.

Parameter estimates and likelihoods were congruent across the four independent chains for models with and without migration (Figures S11–S14). A total of 12 significant migration bands were identified in exploratory analyses and included in the final model (Figure 4b,d). The highest population migration rate (*2Nm*; average number of loci migrating per generation) was found from Sahamamy to Andobo (*2Nm* = 1.15 [95% HPD: 1.09–1.20]) (Table S13), supporting the low genetic differentiation and high connectivity between these populations evident in analyses of population structure. Gene flow among populations south of the Rianila was about one order of magnitude lower, ranging from *2Nm* = 0.11 (95% HPD: 0.10–0.12; Ambodisakoana→Antanambao) to *2Nm* = 0.30 (95% HPD: 0.29–0.32; Antanambao→Ambodisakoana). The remaining migration bands showed even lower rates and accounted for gene flow associated with *M. jollyae* and *M. marohita* as well as between *M. gerpi* populations north and south of the Rianila.

Divergence time estimates differed between the two models and were generally more recent without migration (Figure 4a–c, Table S13), which is in line with findings from Leaché et al. (2014) indicating that the inclusion of migration parameters can increase divergence times. Here, we focus on divergence times estimated under the migration model because a scenario of complete isolation seems unlikely given results of population structure analyses. The migration model suggested that *M. gerpi* diverged from its two sister taxa about 453 ka (95% HPD: 321–603 ka). The split between populations north and south of the Rianila occurred about 254 ka (95% HPD: 179–337 ka), nearly simultaneously with that between *M. marohita* and *M. jollyae* (272 ka [95% HPD: 192–362 ka]). Another parallel divergence was inferred for approximately 40 ka, when Sahamamy and Andobo (43 ka [95% HPD: 29–59 ka]) and Vohiposa/Sahafina and Ambodisakoana (41 ka [95% HPD: 28–54 ka]) split, respectively. Notably, these two splits were not inferred as parallel events under the model without migration, which supported a much younger divergence of Sahamamy and Andobo (3.1 ka [95% HPD: 1.5–4.9 ka]). All divergence events inferred under the migration model coincided with glacial periods (Figure 4c). However, 95% HPD intervals were large, mostly due to uncertainty in generation time and mutation rate.

Absolute estimates of *N*_*e*_ for terminal lineages differed between the two models as well, but relative trends were similar with the exception of Ambodisakoana (Figure 4a,b, Table S13). Effective population sizes ranged from 3600 (95% HPD: 2600–4700) for Andobo to 14,900 (95% HPD: 11,000–19,200) for Vohiposa/Sahafina in the migration model (see Table S13 for corresponding estimates in the model without migration).

Genealogical divergence indices were below 0.2 or within the ambiguity zone for divergences of *M. gerpi* populations located on the same side of the Rianila (Figure 4e, Table S14). In contrast, splits between lineages separated by the river showed an extremely high mean *gdi* of 0.933 (95% HPD: 0.923–0.940; calculated as the mean of pairwise population comparisons), surpassing that of the two sister species *M. jollyae* and *M. marohita* (0.744 [95% HPD: 0.731–0.756]).

### 3.5 Ecological niche modeling

**FIGURE 5:**
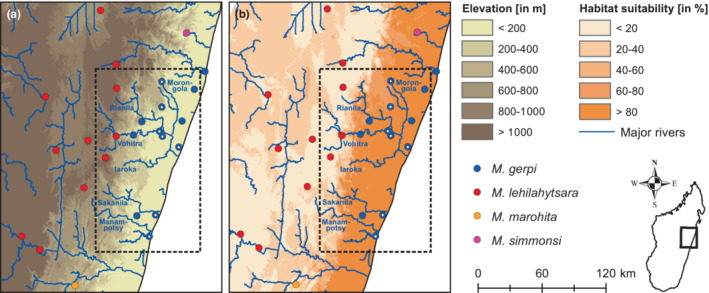
Available sampling localities of *Microcebus gerpi* and adjacent taxa in eastern Madagascar with (a) elevation and (b) habitat suitability for *M. gerpi* inferred under a random forest model in the R package ‘biomod2’. Localities sampled in this study are marked by white dots. The dashed box highlights the area shown in Figure 2.

So far, *M. gerpi* was only found between the Ivondro in the north and the Mangoro in the south (Table S4). Ecological niche models were consistent across approaches and showed that (1) suitable habitats for *M. gerpi* exist beyond these two river barriers limiting their actual distribution, and (2) habitat suitability for *M. gerpi* appears to be negatively correlated with elevation, as high scores (>60%) were only found below 600 m (Figure 5, Figure S15). Due to the inferred admixture at Anjahamana, we defined river size there (i.e., the flow accumulation value generated from a digital elevation model) as the threshold at which rivers did not represent strict barriers for dispersal anymore. This cutoff was applied to all river plots, showing that only around the Morongola at Anjahamana a considerable stretch of suitable habitat can be found, which is not the case for other major rivers in the region due to their larger sizes even at higher elevations (Figure 5, Figure S15).

## 4 DISCUSSION

### 4.1 Rivers, elevation, and paleoclimate shaped the diversification of *M. gerpi*

We investigated the population genomic structure, reconstructed the phylogeographic history, and modeled the ecological niche of *M. gerpi* to identify the role of rivers, elevation, and paleoclimate in diversification processes. We identified strong population structure that was likely shaped by an interaction of these drivers during the diversification of *M. gerpi* in the late Pleistocene. The highest genetic differentiation and deepest divergence was found between populations north and south of the largest river system in the area, the Rianila (~250 ka in the coalescent model with migration; Figure 4b), which is similar to that between its two sister species *M. jollyae* and *M. marohita* (~270 ka). In the southern half of the species distribution, populations separated by rivers also showed reduced connectivity and relatively high genetic differentiation, albeit to a lesser extent than the north–south divide. In contrast, populations on opposite sides of the Morongola and Rongaronga (north of the Rianila) were characterized by high levels of gene flow and migration.

Together with the inferred ecological niche of *M. gerpi*, which appears to be restricted to elevations below 600 m, these findings suggest that riverine barriers were significant contributors to population structure, depending on their size and elevation of headwaters. For instance, the Morongola river does not seem to be a strict barrier for mouse lemurs (at least at its upriver partitions and headwaters), which is supported by the admixed ancestry of individuals from both sides of the river. Niche models showed that at this low altitude high habitat suitability is still provided, which therefore allowed migration between populations of the lowland specialist *M. gerpi*. Rivers separating the southern half of the species distribution (i.e., the Iaroka, Sakanila and Manampotsy) extend further into the highlands (with sizes large enough to function as barriers up to 800 m) and significantly restrict gene flow, although some connectivity remains. Headwaters of the Rianila are located at particularly high elevations (600–1000 m) and exhibit very low habitat suitability for *M. gerpi*. Moreover, this area is probably inhabited by *M. lehilahytsara*, a likely competitor that is not known to occur in sympatry with *M. gerpi*. Taken together, these altitudinal constraints may explain the high differentiation between populations north and south of the river. The barrier function of rivers is further illustrated by contrasting the large potential distribution of *M. gerpi* along the east coast with its actual distribution between the Ivondro and the Mangoro.

Given the timing of diversification, the parallel divergences, and the elevation‐dependent role of riverine barriers restricting connectivity among populations, the phylogeographic history of *M. gerpi* supports a model of diversification through alternating cycles of temporary dispersal around river headwaters and subsequent isolation to refugia during the Pleistocene, as hypothesized by Goodman and Ganzhorn (2004), Mercier and Wilmé (2013), Vences et al. (2009), and Wilmé et al. (2006). Considering present day distributions, the initial divergence between *M. gerpi* and its two sister species likely occurred closer to the southern end of its range, after which it colonized the region between the Ivondro and Mangoro by northward migration. Our data suggest that rivers presented considerable barriers during this process, depending on their size and elevation of headwaters. Some of these, particularly the large Rianila, were likely only traversable by the lowland specialist *M. gerpi* during relatively humid interglacial conditions when suitable vegetation extended to higher elevation regions and provided migration corridors. When conditions grew more arid and cooler during glacials, populations on opposite sides of such rivers became increasingly isolated and started diverging, as they were restricted to vegetational refugia at lower elevations. Indeed, most divergence events in our models overlap with glacial periods (Figure 4c) but confidence in estimates inferred from our coalescent model is limited due to several confounding factors (see below). In the case of the Rianila, a refugium of retained wet conditions was potentially provided between its confluences with the Rongaronga and Iaroka (Figure S16a,b). This area is a natural depression with high values for topographic wetness (Beven & Kirkby, 1979; Figure S16c) and low landscape heterogeneity (Rocchini et al., 2021; Figure S16d). It has been formed most likely due to high levels of discharge accumulating from the entire Vohitra‐Rianila watershed (~7700 km^2^ of surface area, peak at 1472 m) before breaching the coastal mountain ridge through an approximately 660 m wide outlet. Before human cultivation into rice fields, this area must have been an extensive wetland complex, which likely retained enough water during glacial conditions to present a refuge within a less vegetated and dry matrix both north and south of the river. The aforementioned scenario is not only a plausible explanation for the deep divergence between *M. gerpi* populations north and south of the Rianila but can also explain the more recent divergences between populations separated by smaller rivers.

Even complex coalescent models only represent a simplification of the true phylogeographic history of a lineage and can be confounded by several factors. In our final model, uncertainty in parameter estimates is introduced for two main reasons. First, the conversion of *τ* and *θ* to absolute time and effective population size, respectively, introduces considerable uncertainty as it requires estimates of mutation rate and generation time, which are not known exactly for *M. gerpi*. We accounted for this uncertainty by drawing mutation rate and generation time estimates from gamma and lognormal distributions, respectively, leading to relatively wide 95% HPD intervals (Figure 4c). Second, many combinations of gene flow, divergence time and population size may explain the observed genetic variation (i.e., identifiability problem), and it is currently not possible to compare competing models via likelihoods in g‐phocs. For instance, the incorporation of gene flow can affect estimates of *τ* and θ (Leaché et al., 2014; Tseng et al., 2014), which becomes apparent by comparing results of our models with and without migration (Figure 4a–c). Accordingly, careful selection of migration events is crucial for confidence in final parameter estimates. To do so, we ran preliminary models to identify significant migration bands, restricting our analyses to those between recent lineages with geographical proximity to reduce computational burden. We argue that this approach is justified because migration rates were low even for geographically close lineages (except for Sahamamy and Andobo). Not modeling migration between ancient lineages could potentially introduce a larger bias into parameter estimates, but the general timing of diversification in the late Pleistocene is highly supported, and inferred migration rates are concordant with estimated effective migration surfaces, admixture proportions and the neighbornet network (Figure 2a,b, Figures S3, S5–S8). Therefore, we are confident that a diversification model as described in the previous paragraph is robust to these limitations, even though they obstruct the correlation of divergence events with specific glacials and interglacials.

### 4.2 A model for species diversification

The humid rainforests along the east coast are Madagascar's most biodiverse ecoregion, with considerable endemic vertebrate diversity, including lemur species of all extant families, several genera of rodents and carnivores and numerous species of bats, frogs, chameleons, geckos, snakes, skinks, and birds (Crowley, 2004). As mentioned before, several models have been proposed to explain these high levels of biodiversity and endemism, stressing the importance of paleoclimatic oscillations that generated barriers to gene flow by changing water and habitat availability (e.g., Goodman & Ganzhorn, 2004; Mercier & Wilmé, 2013; Wilmé et al., 2006), but empirical support for these hypotheses is limited. Here, we illustrated how such processes can lead to deep divergences within a species by reconstructing the phylogeographic history of *M. gerpi*. The proposed diversification scenario has the potential to explain biodiversity patterns of a variety of humid rainforest taxa, as Madagascar's entire east coast is characterized by eastward flowing rivers on a steep elevational cline, similar to the distributional area of *M. gerpi*. Especially arboreal and terrestrial species are likely limited in their dispersal by similar geographic factors as mouse lemurs, potentially leading to a separation of lineages through rivers and elevation in response to varying paleoclimatic conditions. Depending on the respective time period that passed since colonization of humid lowland habitats, subsequent evolutionary dynamics may range from an initial genetic differentiation and pronounced population structure to complete allopatric speciation (e.g., Pirani et al., 2022). In fact, rivers were already shown to delimit the distributions of frogs (Gehring et al., 2012), lemurs (Goodman & Ganzhorn, 2004; Lei et al., 2017), reptiles and small mammals (Everson et al., 2020) along Madagascar's east coast. However, the underlying diversification processes have rarely been investigated, stressing the need for more phylogeographic studies across diverse taxa. This will also unravel how differences in ecology and dispersal ability made some taxa more resilient and others prone to the isolating effects of paleoclimatically induced barriers to gene flow.

### 4.3 Taxonomic implications

The lack of gene flow, deep divergence and high *gdi* of *M. gerpi* lineages on opposite sides of the Rianila raise the question whether the current taxonomy of *M. gerpi*, *M. marohita* and *M. jollyae* is justified. Following the integrative concept of taxonomy by Padial et al. (2010), genetic distance and reciprocal monophyly alone are insufficient criteria for species delimitation, especially when lineages occur allopatrically. Instead, multiple lines of evidence should be integrated to decide whether (meta‐)populations are evolving independently and can therefore be considered distinct species (sensu de Queiroz, 2007). In the case of *M. gerpi*, preliminary comparisons of morphometric data and ecological niches did not identify significant differences between lineages north and south of the Rianila (Schüßler, Rakotondravony, Radespiel, unpubl. results). Considering this, we do not advocate splitting *M. gerpi* into two species without further supporting evidence such as from ecology or behavior. Rather, our findings highlight the necessity for increased sampling of *M. jollyae* and *M. marohita* to validate their taxonomic status since their descriptions are based on limited sampling and few genetic markers and have not yet been validated by further evidence (Louis et al., 2006; Rasoloarison et al., 2013).

### 4.4 Conservation

*Microcebus gerpi* is currently placed as critically endangered on the IUCN red list of threatened species (Andriaholinirina et al., 2014), based on a single sampling locality at Sahafina forest (Radespiel et al., 2012). Here, we significantly expand knowledge on the distribution and genetic diversity of this species. We show that *M. gerpi* occurs between the Ivondro and Mangoro at elevations up to 600 m. Forests in this region are restricted to small remaining fragments (Figure S15a), and habitat loss and fragmentation will likely continue as deforestation pressures are particularly high in lowland regions that are easy to access (Borgerson et al., 2022; Harper et al., 2007; Schüßler, Mantilla‐Contreras, et al., 2020; Vieilledent et al., 2018). Currently, there are two protected areas in the region, but these are insufficiently small to sustainably maintain *M. gerpi* populations (see Andrianaivoarivelo et al., 2015; Portela et al., 2012). In addition, even in many protected areas deforestation rates remain high due to bureaucratic obstacles and lack of funding (Kappeler et al., 2022). Previous studies have already shown that microendemic mouse lemurs are susceptible to habitat fragmentation (Andriatsitohaina et al., 2020; Schäffler & Kappeler, 2014). Similar to *M. lehilahytsara* (Tiley et al., 2022), *M. gerpi* may be particularly vulnerable to such anthropogenic threats as we identified pronounced population genomic structure originating from paleoclimatic conditions. That is, populations were already highly genetically differentiated and exhibited low levels of gene flow long before human colonization of Madagascar at 2–10 ka (e.g., Dewar et al., 2013; Mitchell, 2019; Pierron et al., 2017). The subsequent anthropogenic habitat loss and fragmentation likely led to considerable isolation of populations and a loss of genetic diversity, as indicated by estimates of *N*_*e*_ (Figure 4a,b) which are at the lower end of those found in populations of other mouse lemur species such as *M. lehilahytsara*, *M. macarthurii*, *M. simmonsi* and *M. jonahi* (Poelstra et al., 2021; Tiley et al., 2022). Given the high degree of ongoing fragmentation of remaining forests in the area, it can be assumed that present‐day gene flow between populations is negligible and that genetic isolation and therefore loss of diversity will increase. Assisted migration within the same IRS could be a measure to mitigate the detrimental effects of inbreeding in fragmented populations while preserving the natural population genetic structure of the species (particularly the high differentiation between northern and southern populations) to avoid potential outbreeding depression (Lynch, 1991). Taken together, our findings provide strong evidence that *M. gerpi* is severely threatened by human activity and could go extinct in the near future if current trends continue.

## AUTHOR CONTRIBUTIONS

**Tobias van Elst:** Conceptualization (equal); data curation (lead); formal analysis (lead); funding acquisition (supporting); investigation (lead); methodology (lead); software (lead); validation (lead); visualization (equal); writing – original draft (lead); writing – review and editing (equal). **Dominik Schüßler:** Formal analysis (supporting); investigation (supporting); methodology (supporting); visualization (equal); writing – original draft (supporting); writing – review and editing (equal). **Romule Rakotondravony:** Funding acquisition (supporting); investigation (supporting); project administration (supporting); resources (equal); writing – review and editing (supporting). **Valisoa S. T. Rovanirina:** Investigation (supporting); writing – review and editing (supporting). **Anne Veillet:** Investigation (supporting); writing – review and editing (supporting). **Paul A. Hohenlohe:** Investigation (supporting); methodology (supporting); resources (equal); writing – review and editing (supporting). **Jonah H. Ratsimbazafy:** Funding acquisition (supporting); project administration (supporting); resources (supporting); writing – review and editing (supporting). **Rodin M. Rasoloarison:** Resources (supporting); writing – review and editing (supporting). **Solofonirina Rasoloharijaona:** Resources (supporting); writing – review and editing (supporting). **Blanchard Randrianambinina:** Resources (supporting); writing – review and editing (supporting). **Miarisoa L. Ramilison:** Investigation (supporting); writing – review and editing (supporting). **Anne D Yoder:** Resources (supporting); writing – review and editing (supporting). **Edward E. Louis, Jr.:** Resources (supporting); writing – review and editing (supporting). **Ute Radespiel:** Conceptualization (equal); data curation (supporting); funding acquisition (lead); investigation (supporting); methodology (supporting); project administration (lead); resources (equal); supervision (lead); validation (supporting); writing – review and editing (equal).

## FUNDING INFORMATION

This work was supported by the German Research Foundation [DFG Ra 502/23‐1 to UR] and through a compute project provided by the North‐German Supercomputing Alliance (HLRN) [nib00015 to UR and TvE]. We also acknowledge the financial support by Global Wildlife Conservation [#5095.008.0175] and Houston Zoo, Inc. [05/GERP/FIN/HSZ‐GERPI/18 to RR and UR]. This Open Access publication was funded by the Deutsche Forschungsgemeinschaft (DFG, German Research Foundation) ‐ 491094227 “Open Access Publication Funding” and the University of Veterinary Medicine Hannover, Foundation.

## CONFLICT OF INTEREST STATEMENT

The authors declare no competing interests.

## Supporting information


Appendix S1

Appendix S2

## Data Availability

All new sequencing data have been made available through NCBI BioProject PRJNA807164. Individual BioSample accessions are given in Table S1. VCF and BEAGLE files, alignments and analyses outputs are available at Dryad (https://doi.org/10.5061/dryad.9w0vt4bmr). Scripts can be found at https://github.com/t‐vane/ResearchSupplements.
