# Supplementary material for: Diversification processes in Gerp's mouse lemur demonstrate the importance of rivers and altitude as biogeographic barriers in Madagascar's humid rainforests
Source: Ecol Evol. 2023 Jul 4;13(7):e10254. doi: 10.1002/ece3.10254 (PMC10318617; doi:10.1002/ece3.10254)
Supplement: Supplementary file 2 — Appendix S2 [file ECE3-13-e10254-s002.pdf]

Diversification processes in Gerp's mouse lemur demonstrate the importance of rivers and altitude as biogeographic barriers in Madagascar's humid rainforests

Supporting Information: Supplementary Methods

Ecology and Evolution

Tobias van Elst[1], Dominik Schüßler[2], Romule Rakotondravony[3,4], Valisoa S. T. Rovanirina[3], Anne Veillet[5], Paul A. Hohenlohe[5], Jonah H. Ratsimbazafy[6], Rodin M. Rasoloarison[7], Solofonirina Rasoloharijaona[3,4], Blanchard Randrianambinina[3,4], Miarisoa L. Ramilison[4,8], Anne D. Yoder[9], Edward E. Louis, Jr.[10], Ute Radespiel[1,*]

[1] Institute of Zoology, University of Veterinary Medicine Hannover, Foundation, Hannover, Germany
[2] Research Group Vegetation Ecology and Nature Conservation, Institute of Biology and Chemistry, University of Hildesheim, Hildesheim, Germany
[3] Ecole Doctorale Ecosystèmes Naturels (EDEN), University of Mahajanga, Mahajanga, Madagascar
[4] Faculté des Sciences, de Technologies et de l'Environnement, University of Mahajanga, Mahajanga, Madagascar
[5] Institute for Bioinformatics and Evolutionary Studies, Department of Biological Sciences, University of Idaho, Moscow, ID, USA
[6] Groupe d'étude et de recherche sur les primates (Gerp), Antananarivo, Madagascar
[7] Behavioral Ecology and Sociobiology Unit, German Primate Center, Göttingen, Germany
[8] Department of Primate Behavior and Ecology, Central Washington University, Ellensburg, WA, USA
[9] Department of Biology, Duke University, Durham, NC, USA
[10] Grewcock Center for Conservation and Research, Omaha's Henry Doorly Zoo and Aquarium, Omaha, NE, USA

* Corresponding author: ute.radespiel@tiho-hannover.de

Supplementary Methods

Genotype filtering

Genotype calls obtained by GATK v4.1.9.0 (McKenna et al., 2010) were filtered following FS6 recommendations of O'Leary et al. (2018) with modified thresholds and using scripts of Poelstra et al. (2021). Unless otherwise mentioned, the filtering was conducted with the options mentioned below in VCFTOOLS v0.1.17 (Danecek et al., 2011). It consisted of the following consecutive steps:

(1) Genotypes with a per-sample depth smaller than 5 were masked ("--minDP 5").

(2) Sites with a mean depth across samples smaller than 5 were removed ("--min-meanDP 5").

(3) Three rounds of filtering sites and individuals based on missing data were applied while excluding monomorphic sites ("--min-allele 2"). Sites were filtered using the "--max-missing" option. Percentage of missing data per individual was calculated using "--missing-indv", and respective individuals were removed with "--remove". The following thresholds for maximum percentage of missing data were applied in this order:

    (1) Site: 50%
    (2) Individual: 90%
    (3) Site: 40%
    (4) Individual: 70%
    (5) Site: 30%
    (6) Individual: 50%

(4) Using VariantAnnotator of GATK v3.8.1 (McKenna et al., 2010), the VCF file was annotated with INFO fields for strandedness of reference vs. alternative allele (FS), root mean square mapping quality (MQ), mapping quality of reference vs. alternative allele (MQRankSum), read position of reference vs. alternative allele (ReadPosRankSum) and allele balance (ABHet). Subsequently, sites satisfying one of the following conditions were set to FILTER using VARIANTFILTRATION of GATK v3.8.1 and removed using VCFTOOLS ("--remove-filtered-all"):

    - FS > 60.0
    - MQ < 40.0
    - MQRankSum < −12.5
    - ReadPosRankSum < −8.0
    - ABHet < 0.2 or ABHet > 0.8

    Detailed information on these INFO fields and threshold recommendations can be found at https://gatk.broadinstitute.org/hc/en-us/articles/360035890471-Hard-filtering-germline-short-variants.

(5) Sites with a mean depth across samples larger than the mean depth across all sites plus twice the standard deviation were removed with the "--max-meanDP" option.

(6) Sites with more than 5% and individuals with more than 45 % of missing data were removed as in step (3).

Genotype likelihood estimation with ANGSD

Genotype likelihoods were estimated with the SAMTOOLS model in ANGSD v.0.934 ("-GL 1") (Korneliussen et al., 2014) following the filtering scheme in Poelstra et al. (2021) while excluding *M. gerpi* individuals that did not pass FS6 filtering and outgroups. In detail, we retained only the following data:

- Sites with a total sequencing depth larger than twice the number of focal individuals and smaller than the sum of the 0.995 quantiles of per-individual sequencing depth distributions ("-setMinDepth 48" and "-setMaxDepth 783.7").
- Sites with an individual depth larger than 2 and smaller than the maximum 0.995 quantile of per-individual sequencing depth distributions ("-setMaxDepthInd 58" and "-setMinDepthInd 2").
- Sites present in at least 75% of focal individuals ("-minInd 37").
- Bases with a quality larger than 20 ("-minQ 20").
- Uniquely mapping and properly paired reads with a minimum mapping quality of 20 ("-uniqueOnly 1", "-only-proper-pairs 1" and "-minMapQ 20")
- Biallelic variants with a probability below 1e-5 ("-skipTriallelic 1" and "-SNP_pval 1e-5").
- Sites with a minor allele frequency (MAF) larger than 0.05 ("-minMaf 0.05"). This filter was only applied for analyses of population structure but not for the inference of MAF spectra.

Locus extraction

We produced FASTA files for phased RAD loci based on filtered genotype calls, using scripts of Poelstra et al. (2021) (see also Poelstra et al., 2022). FASTAALTERNATEREFERENCEMAKER of GATK v3.8.1 was used to produce whole-genome sequences for each individual by substituting called non-reference genotypes in the Mmur3.0 reference. Sites removed during FS6 genotype filtering and sites identified as non-callable by CALLABLELOCI of GATK v3.8.1 (minimum depth of 3) were masked in the resulting FASTA files with BEDTOOLS v2.30.0 (Quinlan & Hall, 2010). Using a custom R script (R Core Team, 2018), stretches composed of at least 25 consecutive called base pairs (non-N) or multiple stretches separated by at most 10 consecutive N were extracted as loci. Subsequently, loci were intersected across individuals, and bases with data for less than 80% of individuals were trimmed from both ends. The data were then filtered to retain only loci with a minimum size of 100 bp, present in at least 90% of focal individuals, and having a minimum inter-locus distance of 10,000 bp. Finally, locus statistics were calculated with AMAS v1.0 (Borowiec, 2016) and loci with more than 5% missing data were removed.

Conversion of coalescent units

Posterior distributions of coalescent units (θ, τ, m) were converted to effective population size (N_e), divergence time in years and population migration rate (*2Nm*) as follows by drawing estimates of generation time from a lognormal distribution with mean ln(3.5) and standard deviation ln(1.16) and estimates of mutation rate (μ) from a gamma distribution with mean 1.236 x 10^{-8} and variance 0.107 x 10^{-8} (see Poelstra et al. 2021):

- N_e = θ / (4 * μ)
- Divergence time in years = (τ * generation time in years) / μ
- *2Nm* = $2Nm_{s \to t}$ = $m_{s \to t} \times \theta_t$ /4, where the subscripts s and t indicate the direction of migration (i.e., from population s to population t)

Supporting Information: Supplementary Figures

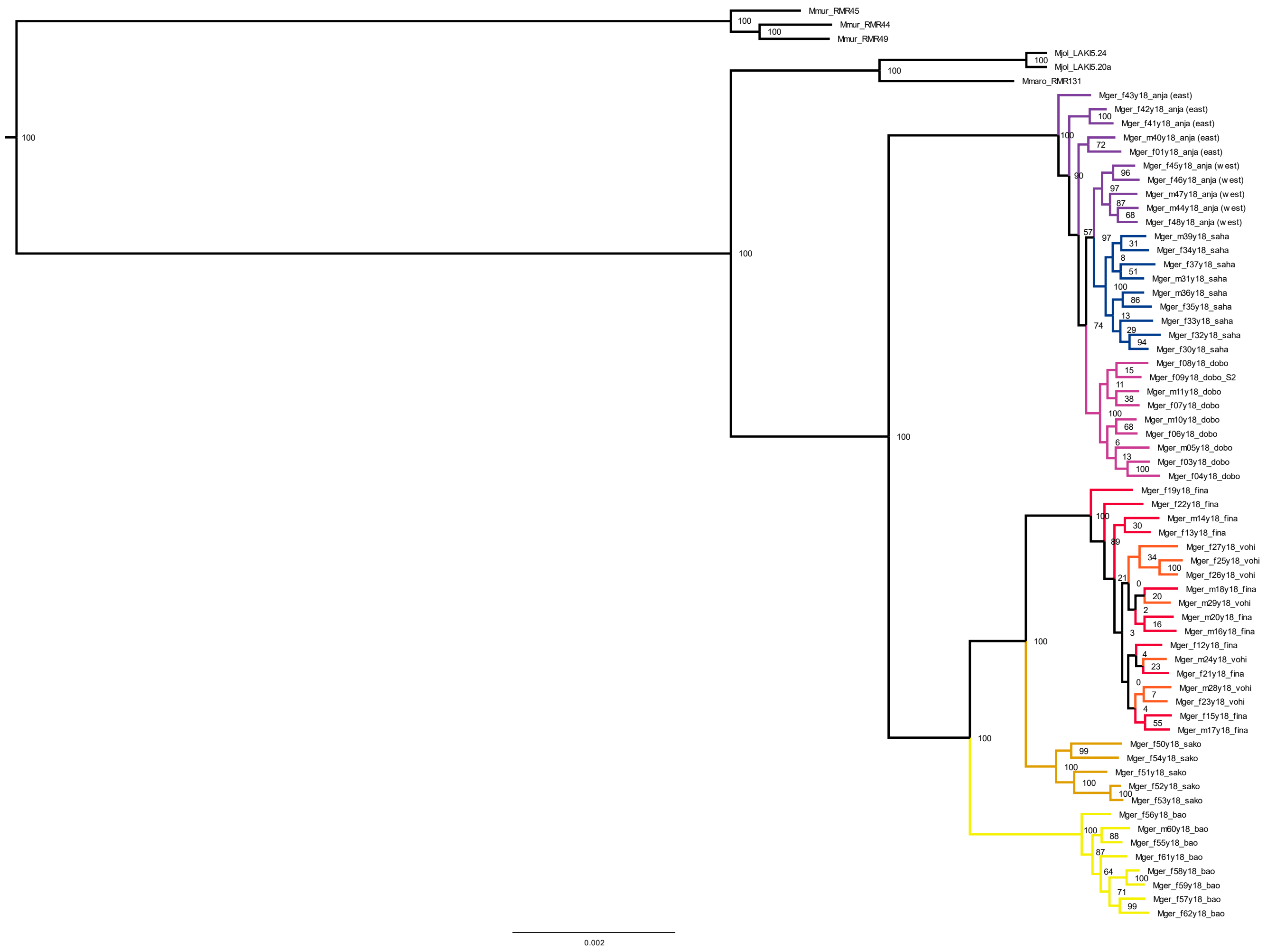

Figure S1: Maximum likelihood phylogeny inferred with RAxML-NG. Scale is in number of substitutions per site. Nodal support represents percent bootstrap (100 replicates). Populations are colored as in Figure 2. For individuals of the population Anjahamana, it is noted which side of the Morongola river they were sampled (east or west).

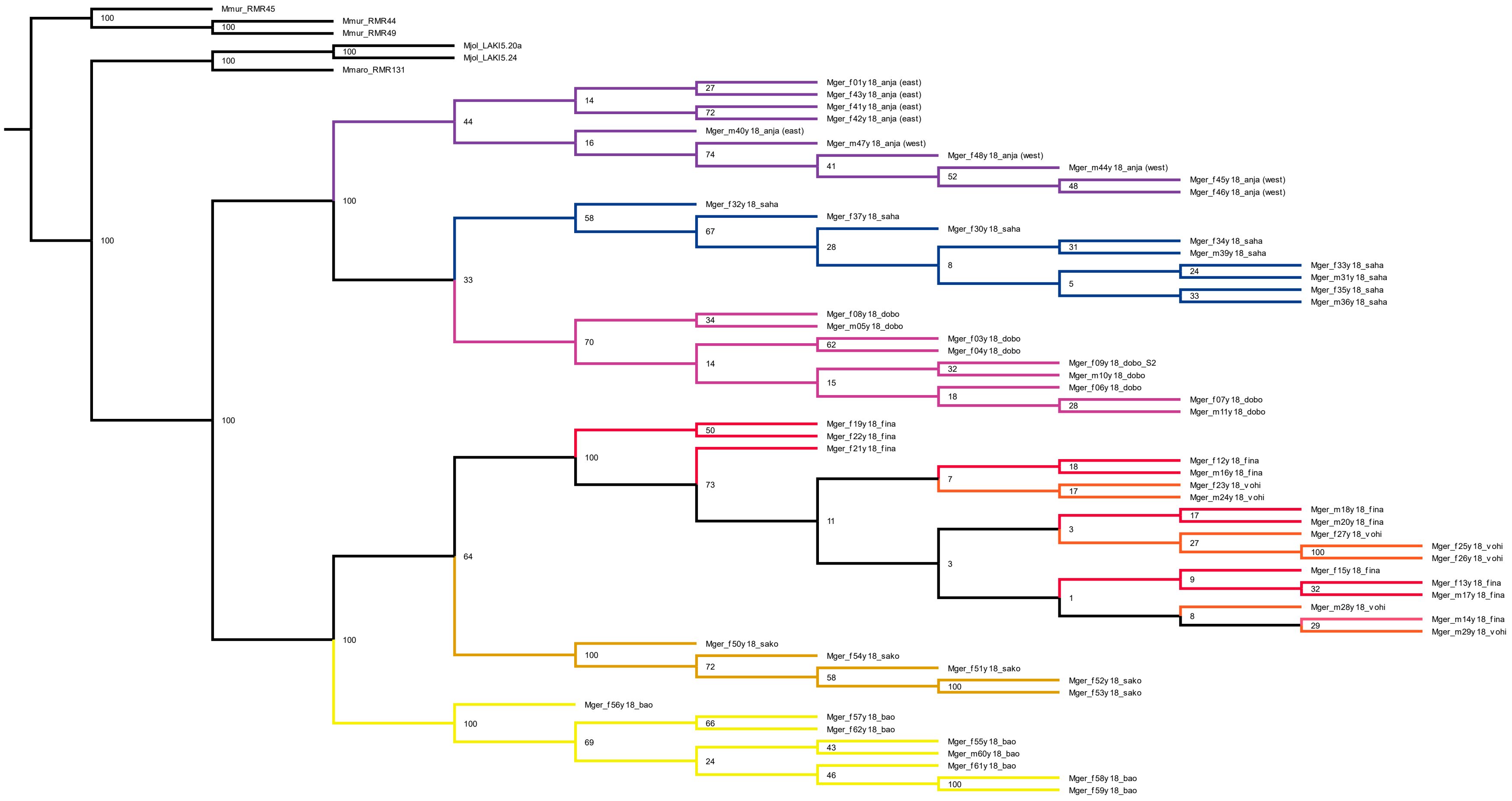

Figure S2: Summary tree inferred with SVDQUARTETS of PAUP*. Single individuals were assigned as tips. Support values from nodes are based on 100 nonparametric bootstraps. Populations are colored as in Figure 2. For individuals of the population Anjahamana, it is noted which side of the Morongola river they were sampled (east or west). Branch lengths contain no information in SVDQUARTETS analyses, and the tree should be interpreted as a cladogram.

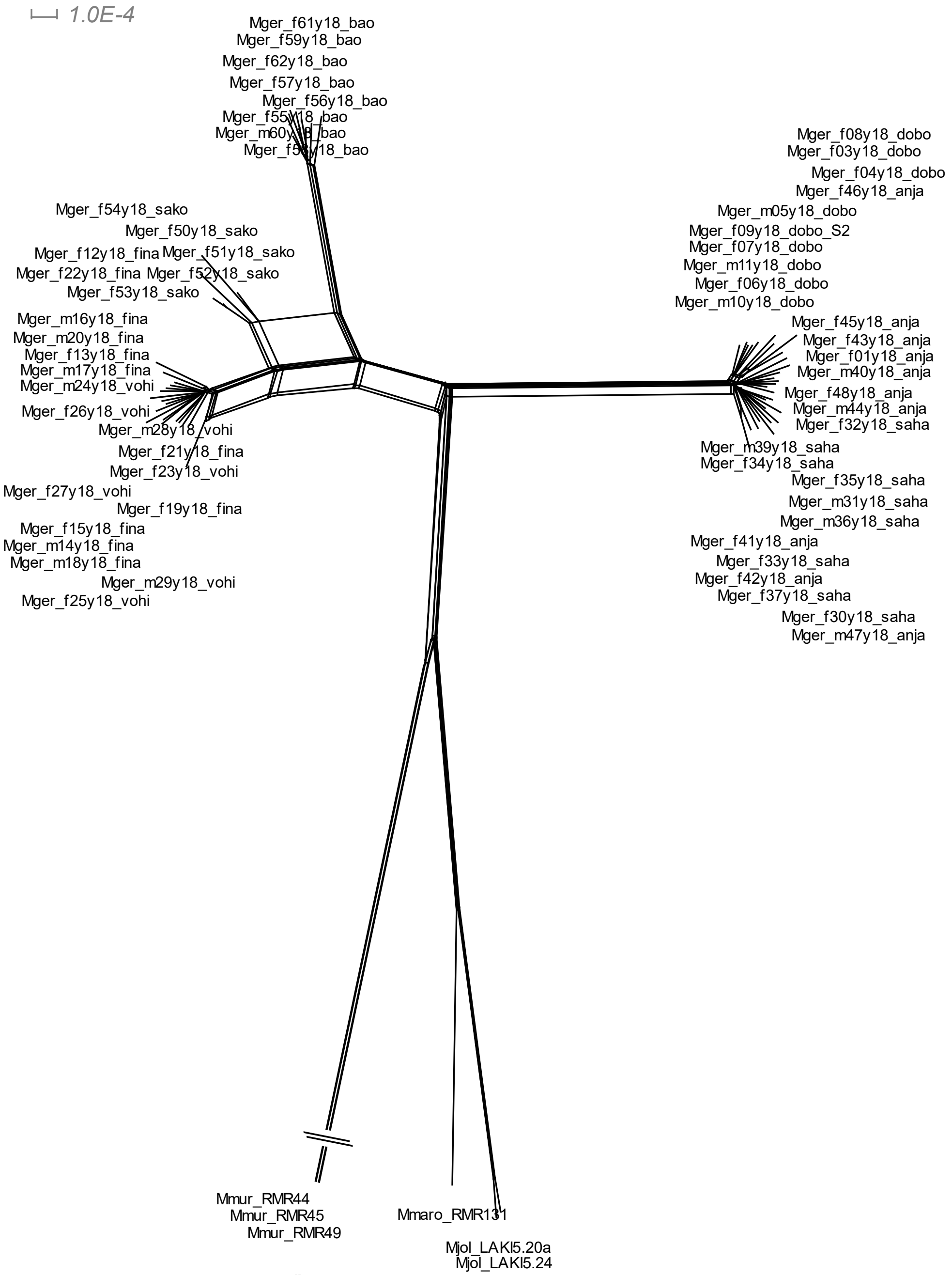

Figure S3: NEIGHBOURNET phylogenetic network estimated with SPLITSTREE.

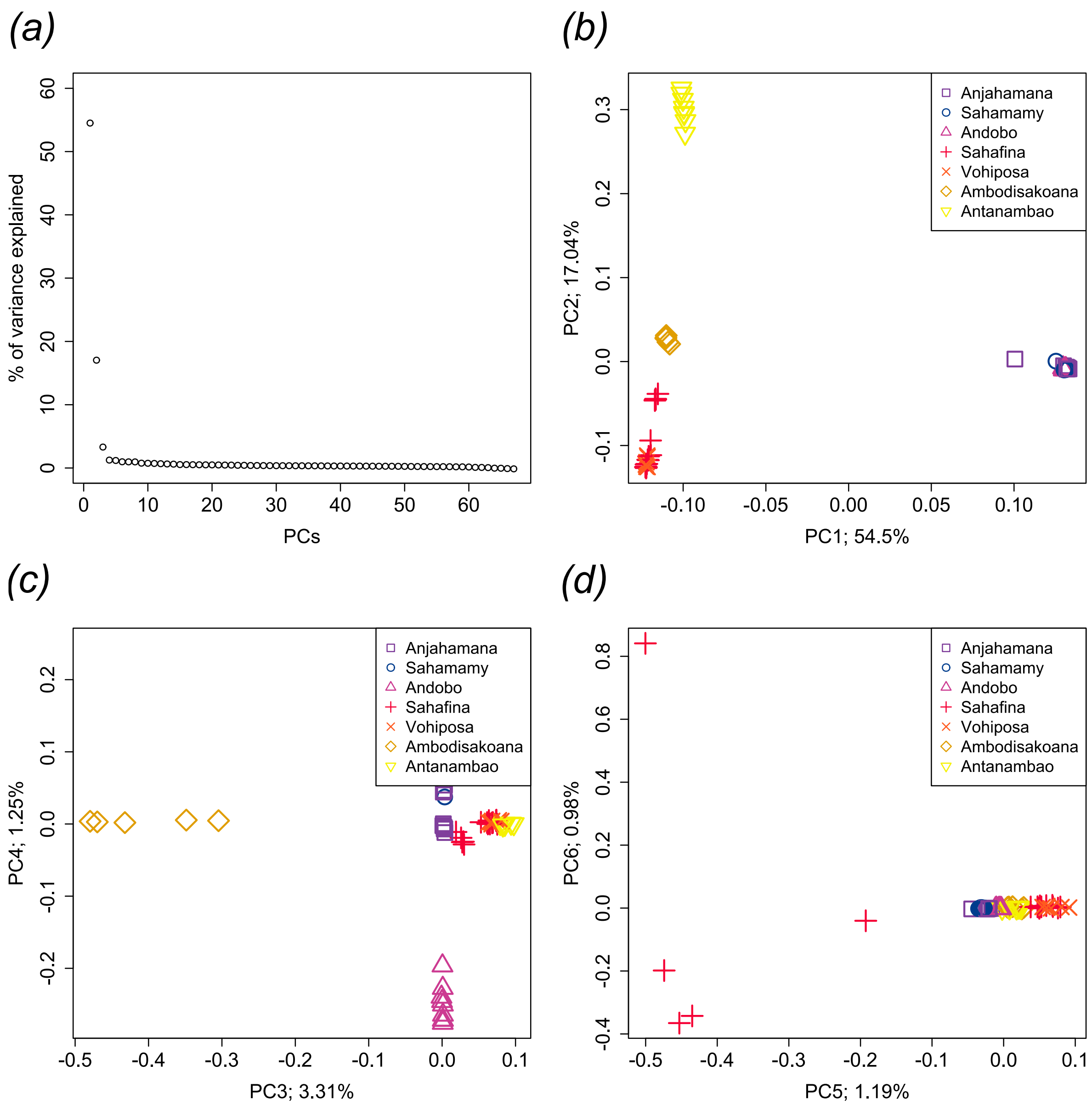

Figure S4: Principal component analyses conducted with PCAngsd. (a) The percentage of variance explained by each principal component (PC). (b) PC1 plotted against PC2. (c) PC3 plotted against PC4. (d) PC5 plotted against PC6.

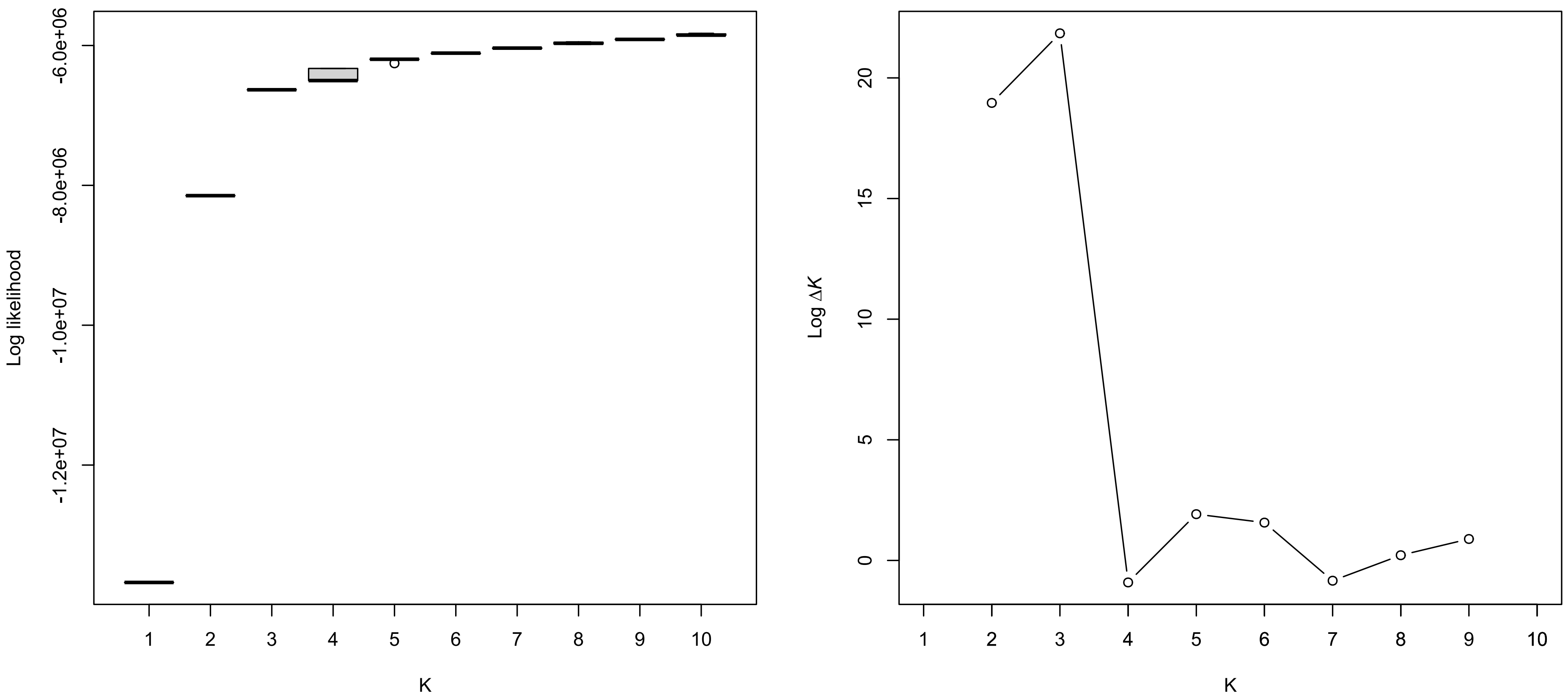

Figure S5: Log-likelihoods and Δ*K* for one to ten *a priori* clusters (*K*) for NGSADMIX analyses. Ten independent runs were conducted for each number of *a priori* clusters.

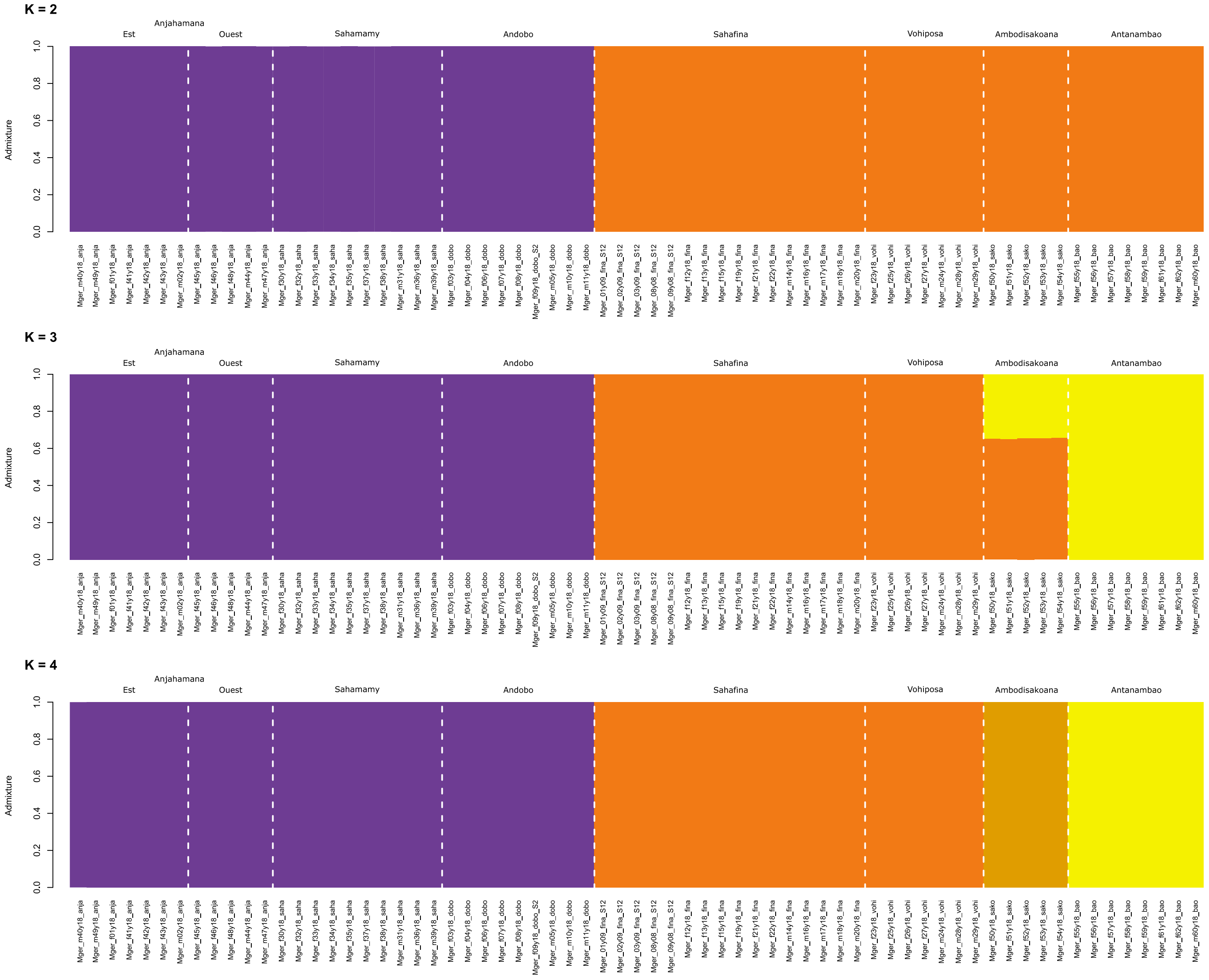

Figure S6: Admixture proportions for two to four *a priori* clusters (*K*) estimated with NGSADMIX. Columns represent ancestries of individuals. Ten independent runs were conducted for each *K*. Shown are the results of the best-scoring likelihood model.

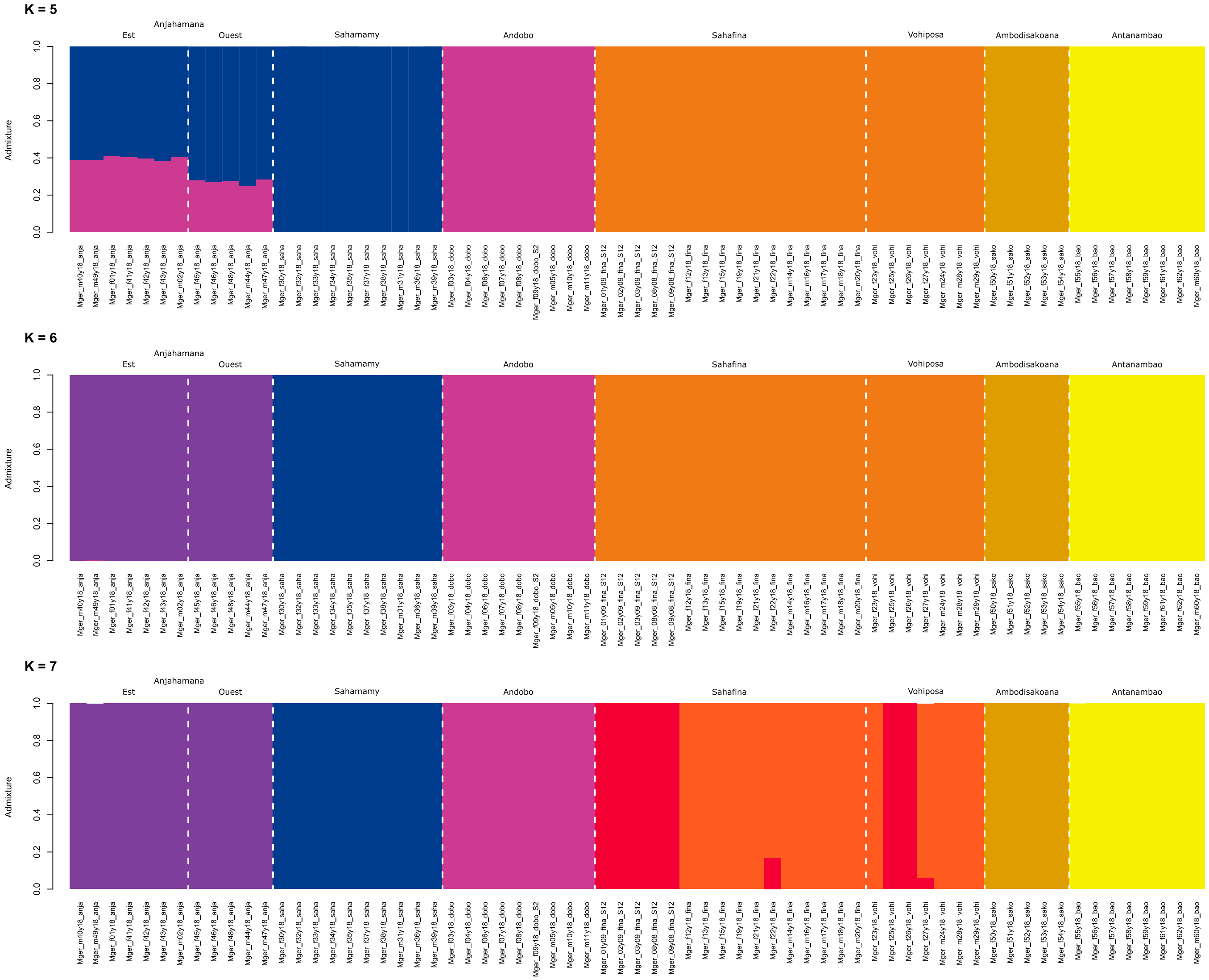

Figure S7: Admixture proportions for five to seven *a priori* clusters (*K*) estimated with NGSADMIX. Columns represent ancestries of individuals. Ten independent runs were conducted for each *K*. Shown are the results of the best-scoring likelihood model.

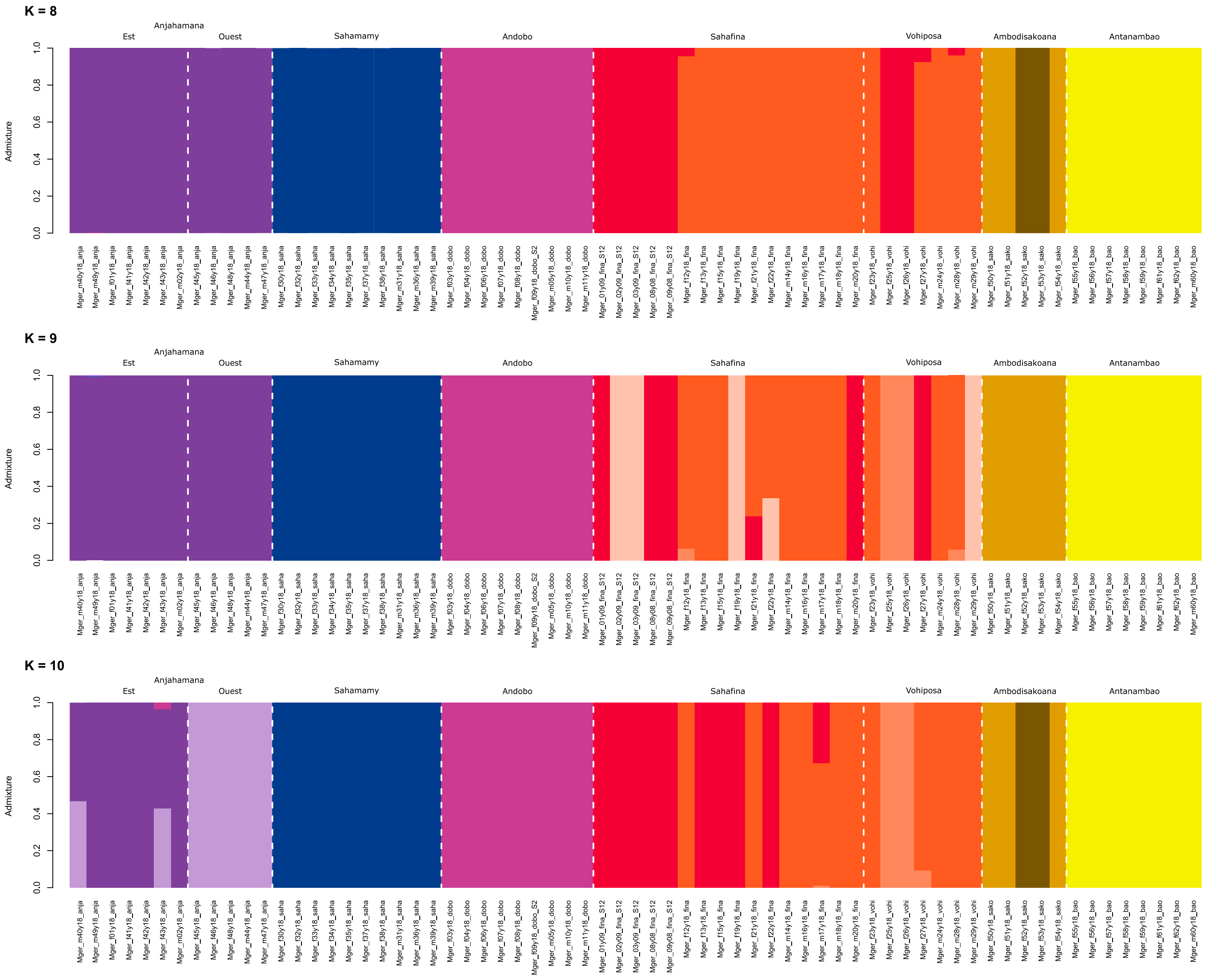

Figure S8: Admixture proportions for eight to ten *a priori* clusters (*K*) estimated with NGSADMIX. Columns represent ancestries of individuals. Ten independent runs were conducted for each *K*. Shown are the results of the best-scoring likelihood model.

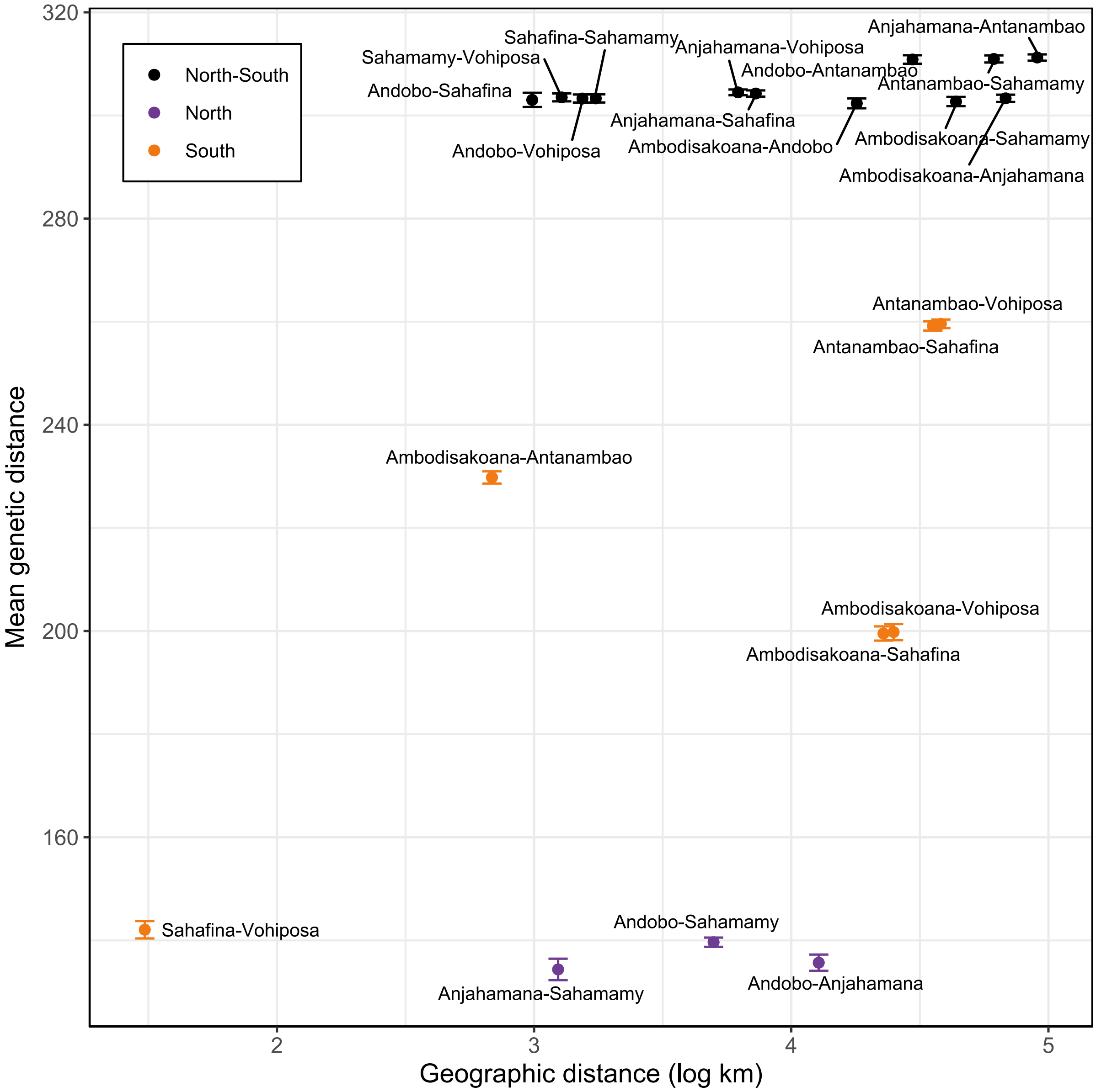

Figure S9: Pairwise genetic distances between *M. gerpi* populations plotted against geographic distances (in km). Genetic and geographic distances between populations were calculated as means between individual distances. Bars show standard deviations of genetic distances. Colors indicate whether the compared populations were north and south of the Rianila (black), both north of the Rianila (purple), or both south of the Rianila (yellow).

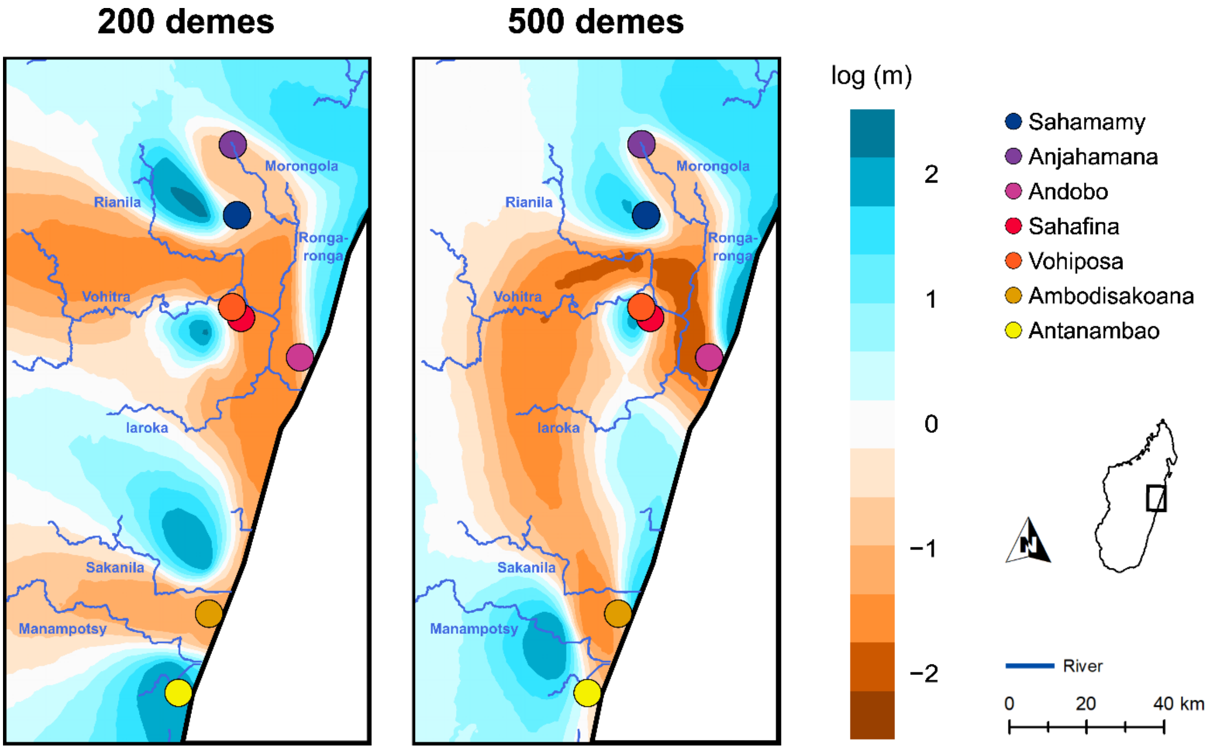

Figure S10: Estimated effective migration surface using 200 and 500 demes. Effective migration rate is given on $\log_{10}$ scale and shown by a color gradient from orange to blue.

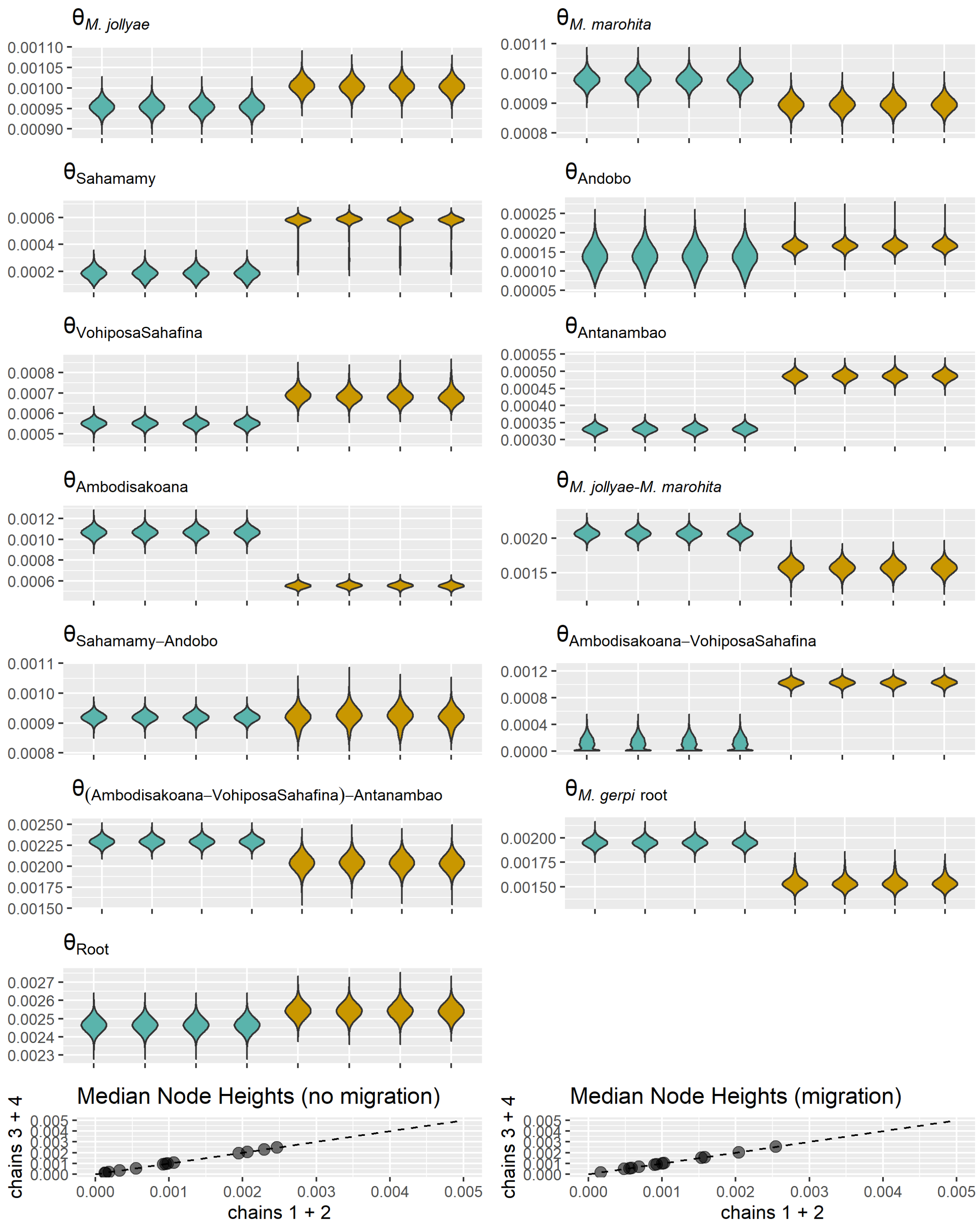

Figure S11: Posterior distributions of θ parameters of G-PhoCS chains. Four independent chains were run for at least 2,000,000 generations with a burn-in of 10% for two alternative models (no migration, green; with migration, yellow), respectively. Combined node heights of chains 1 and 2 were compared to those of chains 3 and 4 to check convergence.

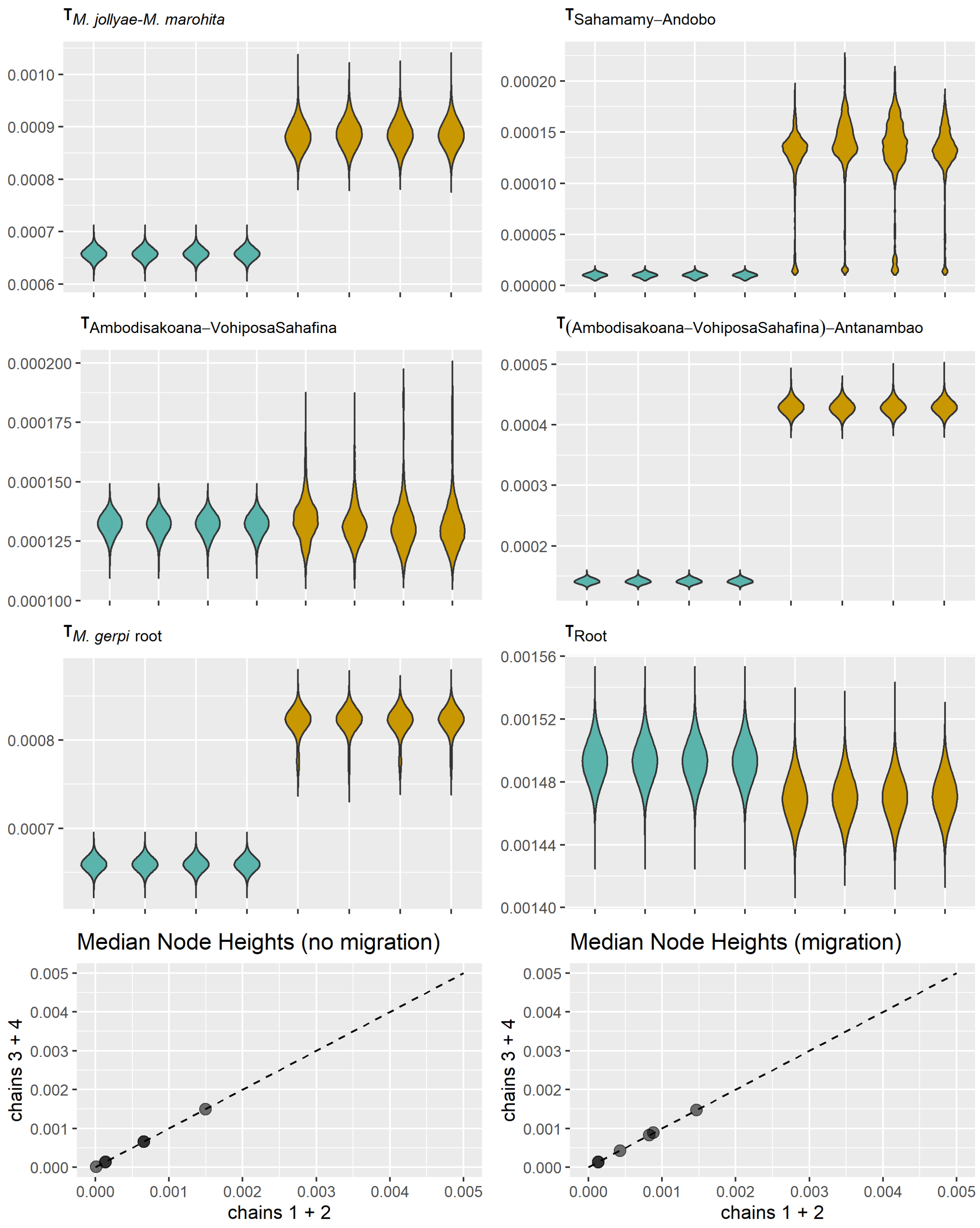

Figure S12: Posterior distributions of *τ* parameters of G-PHOCS chains. Four independent chains were run for at least 2,000,000 generations with a burn-in of 10% for two alternative models (no migration, green; with migration, yellow), respectively. Combined node heights of chains 1 and 2 were compared to those of chains 3 and 4 to check convergence.

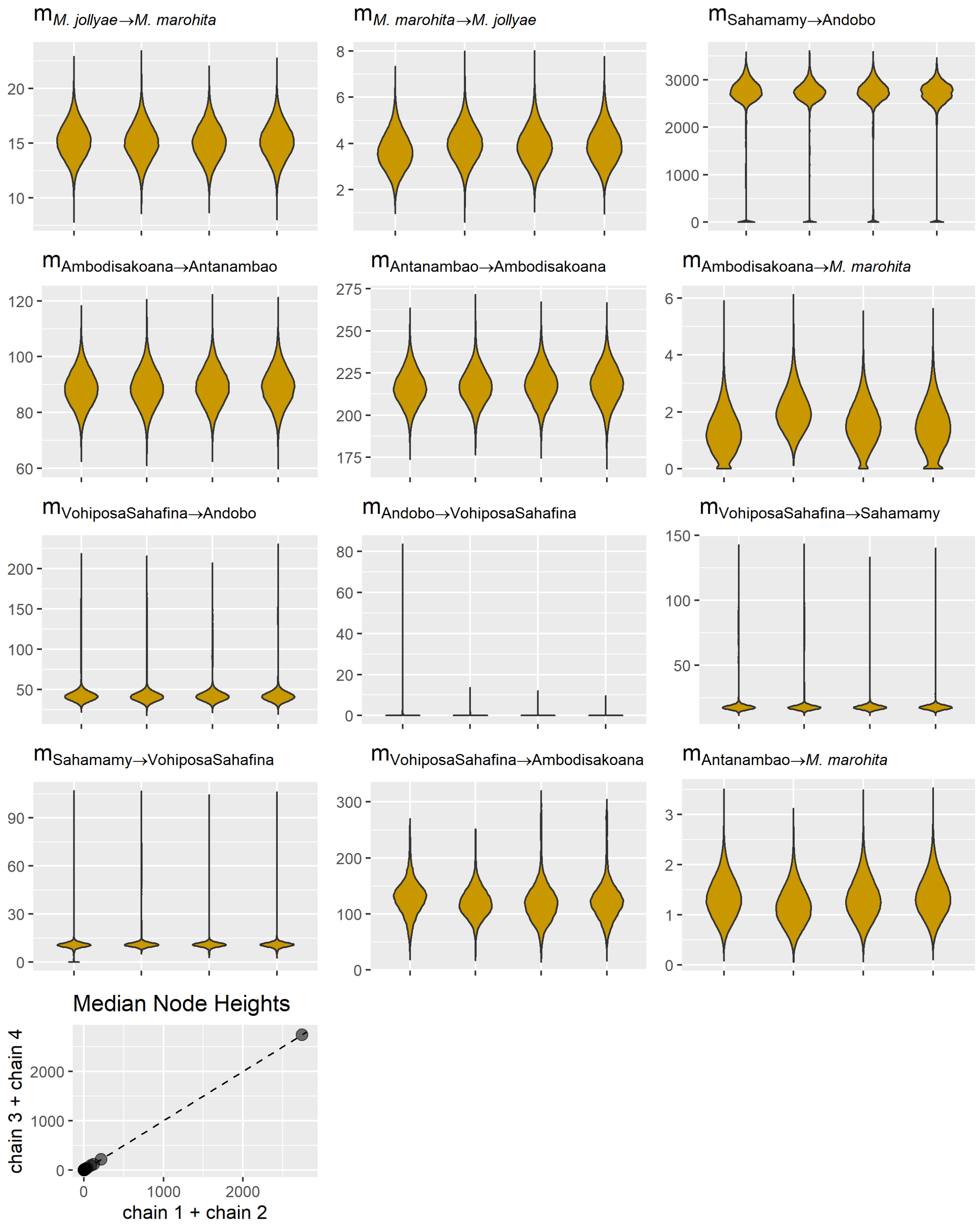

Figure S13: Posterior distributions of migration rate parameters (*m*) of G-PhoCS chains. Four independent chains were run for at least 2,000,000 generations with a burn-in of 10% for the model with migration. Combined node heights of chains 1 and 2 were compared to those of chains 3 and 4 to check convergence.

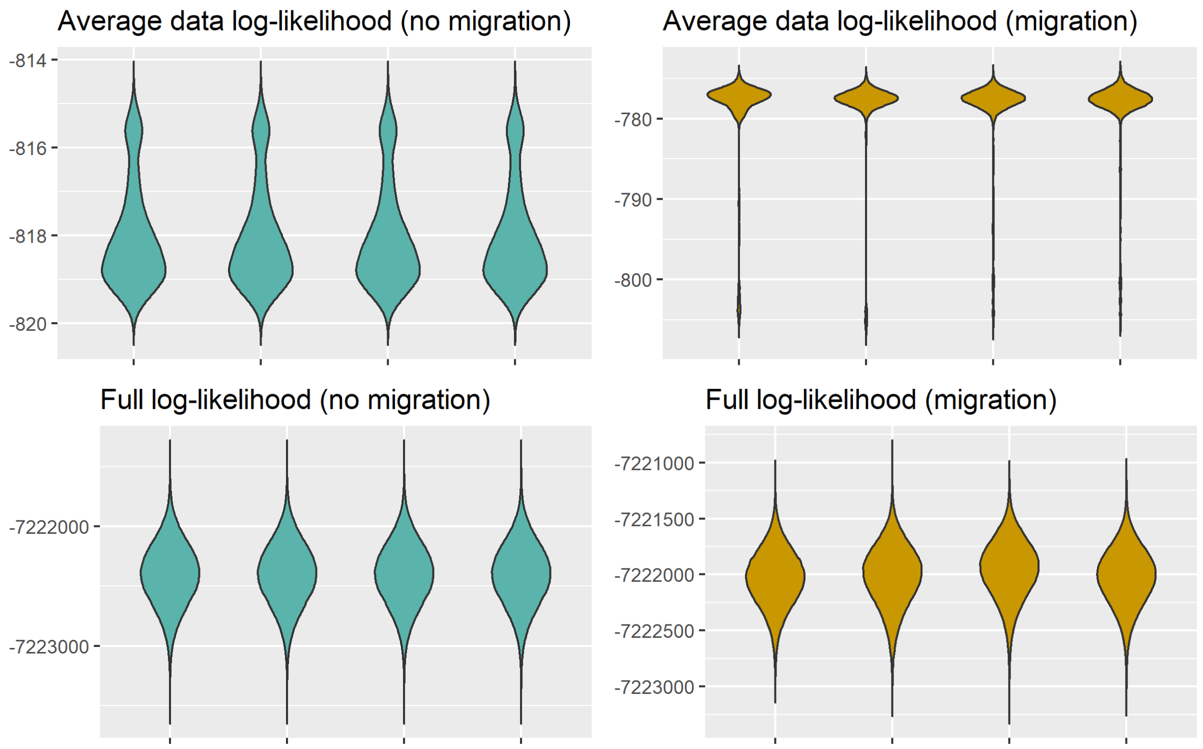

Figure S14: Posterior distributions of average data log-likelihood (across all loci) and full log-likelihood (summed across loci and considering the genealogy priors) of G-PHOCS chains. Four independent chains were run for at least 2,000,000 generations with a burn-in of 10% for two alternative models (no migration, green; with migration, yellow), respectively.

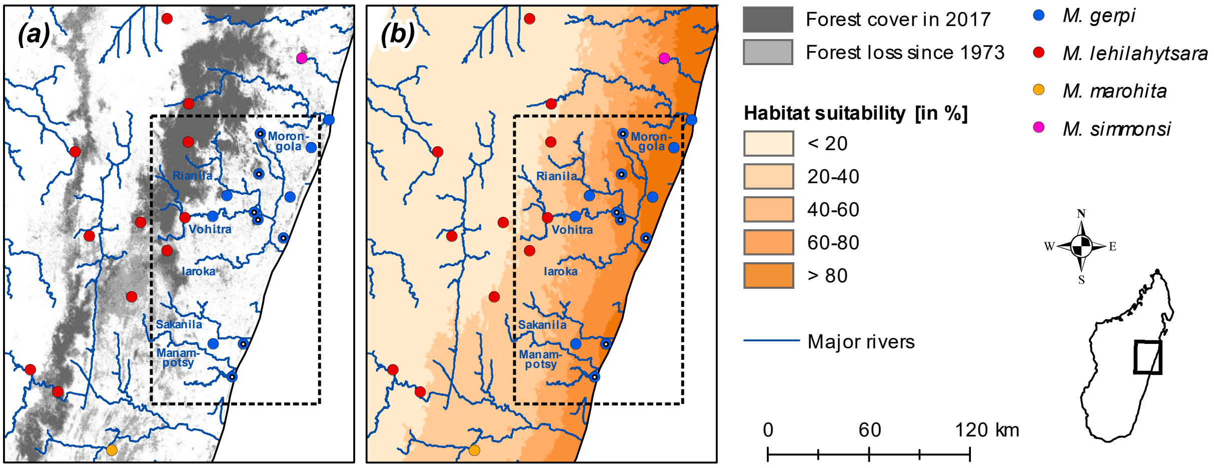

Figure S15: Available sampling localities of *M. gerpi* and adjacent taxa with (a) forest cover change between 1973 and 2017 taken from Schüßler et al. (2020) and Vieilledent et al. (2018) and (b) habitat suitability for *M. gerpi* inferred with the MAXENT algorithm in the R package 'ENMtools'. Localities sampled in this study are marked by white dots. The dashed box highlights the area shown in Figure 2.

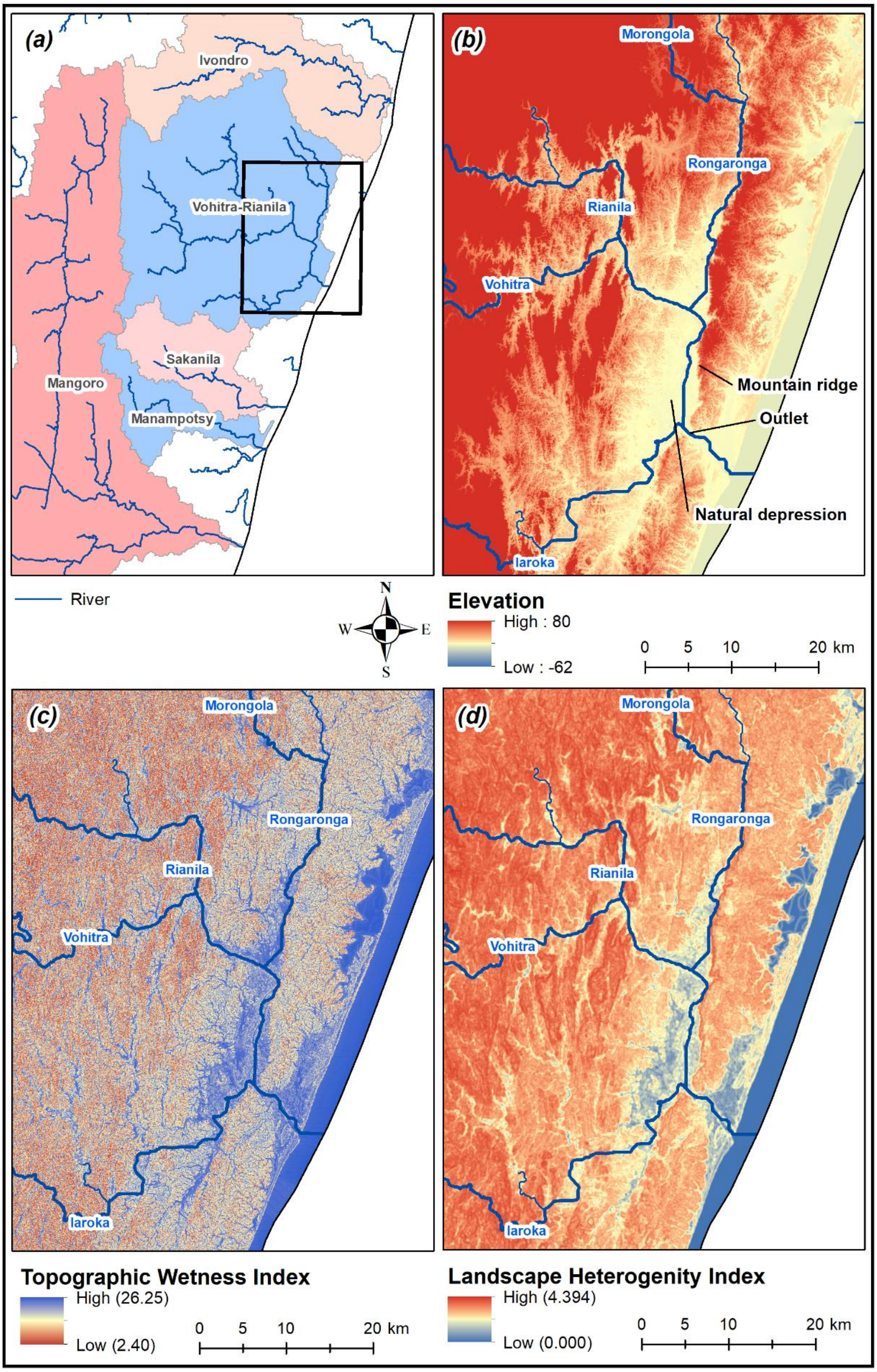

Figure S16: (a) Watersheds of rivers in the distribution of *M. gerpi* including the Rianila (black box) with (b) topography, (c) Topographic Wetness Index (Beven & Kirkby, 1979), and (d) Landscape Heterogeneity Index (Rocchini et al., 2021).

References

Beven, K. J., & Kirkby, M. J. (1979). A physically based, variable contributing area model of basin hydrology / Un modèle à base physique de zone d'appel variable de l'hydrologie du bassin versant. *Hydrological Sciences Journal*, *24*, 43–69. https://doi.org/10.1080/02626667909491834

Borowiec, M. L. (2016). AMAS: a fast tool for alignment manipulation and computing of summary statistics. *PeerJ*, *4*, e1660. https://doi.org/10.7717/peerj.1660

Danecek, P., Auton, A., Abecasis, G., Albers, C. A., Banks, E., DePristo, M. A., ... 1000 Genomes Project Analysis Group. (2011). The variant call format and VCFtools. *Bioinformatics*, *27*, 2156–2158. https://doi.org/10.1093/BIOINFORMATICS/BTR330

Korneliussen, T. S., Albrechtsen, A., & Nielsen, R. (2014). ANGSD: Analysis of next generation sequencing data. *BMC Bioinformatics*, *15*, 356. https://doi.org/10.1186/s12859-014-0356-4

McKenna, A., Hanna, M., Banks, E., Sivachenko, A., Cibulskis, K., Kernytsky, A., ... DePristo, M. A. (2010). The genome analysis toolkit: A MapReduce framework for analyzing next-generation DNA sequencing data. *Genome Research*, *20*, 1297–1303. https://doi.org/10.1101/gr.107524.110

O'Leary, S. J., Puritz, J. B., Willis, S. C., Hollenbeck, C. M., & Portnoy, D. S. (2018). These aren't the loci you'e looking for: Principles of effective SNP filtering for molecular ecologists. *Molecular Ecology*, *27*, 3193–3206. https://doi.org/10.1111/mec.14792

Poelstra, J. W., Montero, B. K., Lüdemann, J., Yang, Z., Rakotondranary, S. J., Hohenlohe, P., ... Yoder, A. D. (2022). RADseq data reveal a lack of admixture in a mouse lemur contact zone contrary to previous microsatellite results. *Proceedings of the Royal Society B*, *289*. https://doi.org/10.1098/RSPB.2022.0596

Poelstra, J. W., Salmona, J., Tiley, G. P., Schüßler, D., Blanco, M. B., Andriambeloson, J. B., … Yoder, A. D. (2021). Cryptic patterns of speciation in cryptic primates: Microendemic mouse lemurs and the multispecies coalescent. *Systematic Biology*, *70*, 203–218. https://doi.org/10.1093/sysbio/syaa053

Quinlan, A. R., & Hall, I. M. (2010). BEDTools: A flexible suite of utilities for comparing genomic features. *Bioinformatics*, *26*, 841–842. https://doi.org/10.1093/bioinformatics/btq033

R Core Team. (2018). *R: A language and environment for statistical computing* (3.5.1). R Foundation for Statistical Computing.

Rocchini, D., Thouverai, E., Marcantonio, M., Iannacito, M., Da Re, D., Torresani, M., … Wegmann, M. (2021). rasterdiv — An information theory tailored R package for measuring ecosystem heterogeneity from space: to the origin and back. *Methods in Ecology and Evolution*, *12*, 1093–1102. https://doi.org/10.1111/2041-210X.13583

Schüßler, D., Mantilla-Contreras, J., Stadtmann, R., Ratsimbazafy, J., & Radespiel, U. (2020). Identification of crucial stepping stone habitats for biodiversity conservation in northeastern Madagascar using remote sensing and comparative predictive modeling. *Biodiversity and Conservation*, *29*, 2161–2184. https://doi.org/0.1007/s10531-020-01965-z

Vieilledent, G., Grinand, C., Rakotomalala, F. A., Ranaivosoa, R., Rakotoarijaona, J. R., Allnutt, T. F., & Achard, F. (2018). Combining global tree cover loss data with historical national forest cover maps to look at six decades of deforestation and forest fragmentation in Madagascar. *Biological Conservation*, *222*, 189–197. https://doi.org/10.1016/j.biocon.2018.04.008
